# Multi LC-MS/MS and LC-HRMS Methods for Determination of 24 Mycotoxins including Major Phase I and II Biomarker Metabolites in Biological Matrices from Pigs and Broiler Chickens

**DOI:** 10.3390/toxins11030171

**Published:** 2019-03-19

**Authors:** Marianne Lauwers, Siegrid De Baere, Ben Letor, Michael Rychlik, Siska Croubels, Mathias Devreese

**Affiliations:** 1 Department of Pharmacology, Toxicology and Biochemistry, Faculty of Veterinary Medicine, Ghent University, 9820 Merelbeke, Belgium; marianne.lauwers@ugent.be (M.L.); siegrid.debaere@ugent.be (S.D.B.); siska.croubels@ugent.be (S.C.); 2 Innovad, Postbaan 69, 2910 Essen, Belgium; b.letor@innovad-global.com; 3 Chair of Analytical Food Chemistry, Technische Universität München, Maximus-von-Imhof-Forum 2, 85354 Freising, Germany; michael.rychlik@tum.de

**Keywords:** Biomarkers, exposure, LC-MS/MS, LC-HRMS, pig, broiler chicken, multi-mycotoxin

## Abstract

A reliable and practical multi-method was developed for the quantification of mycotoxins in plasma, urine, and feces of pigs, and plasma and excreta of broiler chickens using liquid chromatography–tandem mass spectrometry. The targeted mycotoxins belong to the regulated groups, i.e., aflatoxins, ochratoxin A and *Fusarium* mycotoxins, and to two groups of emerging mycotoxins, i.e., *Alternaria* mycotoxins and enniatins. In addition, the developed method was transferred to a LC-high resolution mass spectrometry instrument to qualitatively determine phase I and II metabolites, for which analytical standards are not always commercially available. Sample preparation of plasma was simple and generic and was accomplished by precipitation of proteins alone (pig) or in combination with removal of phospholipids (chicken). A more intensive sample clean-up of the other matrices was needed and consisted of a pH-dependent liquid–liquid extraction (LLE) using ethyl acetate (pig urine), methanol/ethyl acetate/formic acid (75/24/1, *v*/*v*/*v*) (pig feces) or acetonitrile (chicken excreta). For the extraction of pig feces, additionally a combination of LLE using acetone and filtration of the supernatant on a HybridSPE-phospholipid cartridge was applied. The LC-MS/MS method was in-house validated according to guidelines defined by the European and international community. Finally, the multi-methods were successfully applied in a specific toxicokinetic study and a screening study to monitor the exposure of individual animals.

## 1. Introduction

The worldwide contamination of feed with mycotoxins is of major agro-economic importance. In addition to crop and feed loss and damage, these mycotoxins can have a large impact on animal health. Surveys show that mycotoxins occur in more than 70% of the tested feed samples and 38% of these samples contain multiple mycotoxins. Co-contamination of several mycotoxins can result in additive or synergistic effects. Consequently, multi-methods are an asset in mycotoxin analysis [1].

This study focused on the mycotoxins regulated by the European Union [2,3], as well as the mycotoxins for which legislation is currently lacking but which reveal evident toxicity and high prevalence in feed [4]. More specifically, aflatoxin B1 (AFB1), ochratoxin A (OTA), fumonisin B1 (FB1), T2-toxin (T2), zearalenone (ZEN) and deoxynivalenol (DON), as well as enniatins (ENN), beauvericin (BEA), alternariol (AOH) and tenuazonic acid (TeA) were included.

Traditionally, mycotoxins are determined and regulated at the level of the feed. However, feed analysis has some major disadvantages. First, the possible presence of mycotoxin hotspots, i.e., local areas in the feed with a higher concentration, can cause an unequal distribution of mycotoxins in the feed and make it difficult to obtain a representative sample [5]. Second, analyzing feed gives no information about the individual exposure. Fluctuations can arise from differences in food consumption or in absorption, distribution, biotransformation and excretion (ADME) processes between the animals. Third, the risk associated with exposure can be underestimated because feed analysis does not include alternative routes of exposure such as dermal and respiratory exposure [6,7]. Fourth, modified or conjugated forms, previously called masked mycotoxins, can convert back to their free forms and hence contribute to the adverse effects related to mycotoxin exposure. This has been demonstrated for 3- and 15-acetyldeoxynivalenol (3ADON and 15ADON, respectively) and DON-3-glucoside (DON3G) in pigs and broiler chickens [8,9]. Therefore, both ADONs and DON3G can be considered as toxic as DON itself. Detection of these modified forms in feed can be difficult and is not always possible with conventional methods where the non-modified mycotoxin is determined. This creates a possible mismatch between the feed contamination level and the exposure of the animals [10]. Finally, clinical signs of mycotoxin exposure can appear when the contaminated feed has already been consumed, thus complicating or preventing diagnosis of herd problems associated with mycotoxins [11].

These issues can be resolved by biomonitoring of the animals and determining the exposure to mycotoxins in biological matrices with the use of so-called biomarkers of exposure. Biomarkers are molecules related to the exposure and are often the mycotoxin itself, the in vivo formed metabolites or interaction products with macromolecules such as nucleic acids or proteins [12,13].

The selected mycotoxins and their phase I and II metabolites or interaction products (e.g., aflatoxin-guanine) can be measured in several biological matrices. In this study, plasma, urine and feces of pigs and plasma and excreta of broiler chickens were chosen as biological matrix since combining these enables studying all the in vivo toxicokinetic parameters and the complete metabolic profile. Moreover, these matrices can be used to determine the efficacy of mycotoxin detoxifiers according to the guidelines of the European Food Safety Authority (EFSA) [14]. In addition, they can be easily applied for the detection of mycotoxins in these animals under field conditions. Especially, the non-invasive character of urine and feces collection can be of added value when sampling on farm.

Nowadays, state-of-the-art equipment such as liquid chromatography (LC) coupled to a tandem mass spectrometer (MS/MS) or a high-resolution mass spectrometer (HRMS) have become the standard in determining mycotoxins in biological matrices. Indeed, several LC-MS/MS multi-methods (≥2 mycotoxins) have already been developed for the determination of mycotoxins in biological matrices of pigs and broiler chickens. However, most of these multi-methods are limited to one group of mycotoxins. Such methods are available for (the metabolites of) DON [9,15,16,17,18], ZEN [19,20], ENNs [21,22], T2 [23,24,25], AFB1 [26] and TeA [27]. Only few multi-methods combine mycotoxins from different families. To the best of our knowledge, this is the case for urine [28,29,30,31,32,33] and plasma [33,34,35] of pigs, and plasma [35] of broiler chickens. These multiclass methods are also available for other animal species such as for fish plasma [36], human plasma and urine [5,37,38,39,40], and rat plasma and urine [39]. The methods for urine clean-up often apply immunoaffinity columns or solid phase columns [30,31,32,33]. This approach increases the sensitivity but also the cost of analyzing a large number of samples and it limits the number of analytes that can be detected. To avoid these limitations, the dilute and shoot approach without further pre-treatment is frequently used [5,37,39,40]. However, this method demands careful optimization of the dilution factor, and often matrix effects and interfering matrix peaks are observed, which might decrease the sensitivity. Therefore, liquid–liquid extractions (LLE) are preferred as urine sample preparation because they are easy to perform in large quantity and enough sensitivity can be obtained. The developed plasma methods by Brezina et al. [41] and De Baere et al. [35] use OASIS HLB columns that require conditioning before use, limiting the number of samples that can be processed in a period of time. To increase the possible number of samples processed, LLE and protein precipitation are preferred. This study therefore aimed to develop a practical, fast and cost-efficient multi-method for analysis of plasma and urine of pigs and plasma of chickens, using LLE and protein precipitation, avoiding the use of affinity/solid phase columns.

Multi-methods are to the best of our knowledge currently not available for pig feces and excreta of broiler chickens. This might be due to high matrix complexity and the diversity of the physicochemical characteristics of the mycotoxins. Hence, the aim of this study was also to address these difficulties and to develop a multi-method in these highly complex matrices.

LC-HRMS is especially interesting to elucidate and determine phase I and II metabolites and interaction products for which analytical standards are not easily available. Especially, since phase II metabolites might be the most appropriate biomarkers for some mycotoxins due to the extensive biotransformation by these pathways. The glucuronidated and sulfated metabolites of DON and ZEN, as well as the metabolites formed after hepatic biotransformation of ENNB and B1 have already been determined using LC-HRMS [9,20,22,42]. However, to our knowledge, these metabolites of several toxins belonging to multiple classes have never been determined in a single chromatographic run using LC-HRMS.

Thus, the combination of LC-MS/MS and LC-HRMS enables not only determining mycotoxins with an appropriate sensitivity in the lower ng/mL or ng/g range but also detecting a broad range of mycotoxins, i.e., the mycotoxins and metabolites for which standards are readily available as well as other phase I and II metabolites and interaction products. This is especially interesting when assessing the most appropriate biomarker for exposure.

Therefore, the aim of this study was to develop and validate multi-methods using LC-MS/MS to determine relevant mycotoxins in biological matrices of pigs and broiler chickens. In addition, the LC-MS/MS method was transferred to LC-HRMS to determine mycotoxins for which analytical standards are not always commercially available. It is important to consider the main challenges including the high complexity of the matrix, the large range of different physicochemical characteristics of the mycotoxins and the need for a method with an appropriate sensitivity (in the lower ng/mL or ng/g range), so that they can be used not only for specific toxicokinetic studies but also for screening studies to monitor the exposure of individual animals.

## 2. Results and Discussion

### 2.1. Method Development

#### 2.1.1. Sample Preparation and Extraction

Three requirements were prioritized in the development of adequate sample pre-treatments for the different matrices. First, the sample preparation should be simple and practical, thereby enabling analysis of a large quantity of samples in a time and cost efficient way. Second, the sample preparation should be as generic as possible, to allow the extraction of the 24 mycotoxins including some relevant metabolites, which was a real challenge taking into account the various physicochemical properties of the different classes of mycotoxins. Third, the method should be sensitive and, therefore, an LOQ of 1 ng/mL of ng/g was aimed for all analytes in all matrices.

**Figure 1:**
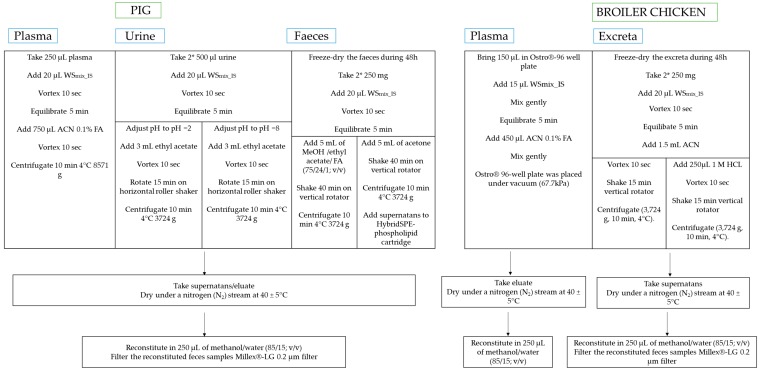
Flowchart of the final methods to determine different mycotoxins in plasma, urine and feces of pigs, and plasma and excreta of broiler chickens.

Initially, all methods started from the most generic and simple sample preparation: deproteinization with an organic solvent. However, due to high matrix complexity and the large variety in physicochemical characteristics of the different mycotoxins more complex methods were needed for urine, feces and excreta. A summary of the final protocols can be found in the flowchart below (Figure 1).

##### Pig and Chicken Plasma

Method development was started with the optimization of the extraction of the analytes of interest from pig and chicken plasma. Proteins and phospholipids are often removed from plasma samples before analysis on LC-MS/MS equipment to prevent clotting and contamination of the equipment. Deproteinization can be accomplished using organic solvents, such as MeOH and ACN. The elimination of phospholipids could be obtained using an Oasis Ostro^®^-plate or HybridSPE^®^-phospholipid 30 mg/1 mL solid-phase extraction (SPE) tubes. The use of the Ostro^®^-plate required the addition of 0.1% formic acid. Therefore, 0.1% formic acid was added to ACN and MeOH.

For pig plasma, deproteinization with 0.1% formic acid in MeOH and 0.1% formic acid in ACN were tested in triplicate on spiked pig plasma samples (analyte concentration: 10 ng/mL). In this study, ACN was preferred as deproteinization solvent for pig plasma, compared to MeOH, since it gave a clearer supernatant after centrifugation. This is in accordance with previous studies where deproteinization with ACN was successfully applied to detect mycotoxins in pig plasma [20,34].

**Figure 2:**
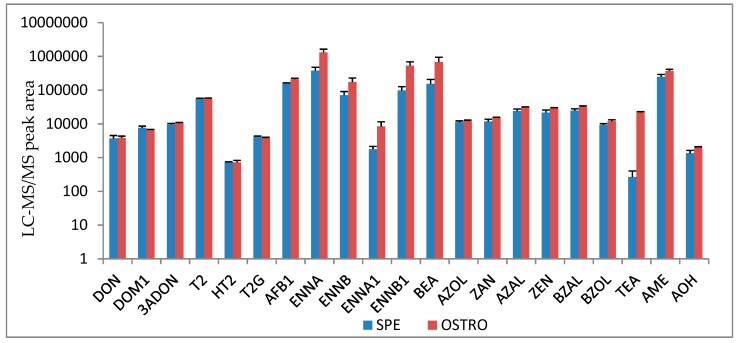
Comparison of the chromatographic peak areas of the different mycotoxins (mean + SD) after extraction from broiler chicken plasma (spiked at 10 ng/mL) using Oasis Ostro^®^ plate or hybrideSPE^®^ phospholipid SPE tubes (*n* = 3 per protocol).

Plasma of broiler chickens contains more phospholipids compared to pigs [43]. Therefore, an additional removal of the phospholipids was necessary to obtain clear samples and to prevent clogging of tubings of the LC-MS/MS and LC-HRMS instruments during routine sample analysis. Spiked broiler chicken plasma samples (analyte concentration: 10 ng/mL) were extracted using the Oasis Ostro^®^-plate or the hybrideSPE^®^ phospholipid SPE tubes (*n* = 3 per protocol). As can be seen in Figure 2, the use of SPE tubes resulted in a lower peak area, especially for ENNs, BEA, TEA, AME and AOH. Consequently, for broiler chicken plasma, deproteinization with ACN and 0.1% formic acid was combined with the Oasis Ostro^®^-plate to obtain the best results. This combination was already successfully applied to detect mycotoxins in chicken plasma by our group [44].

##### Pig Urine

**Figure 3:**
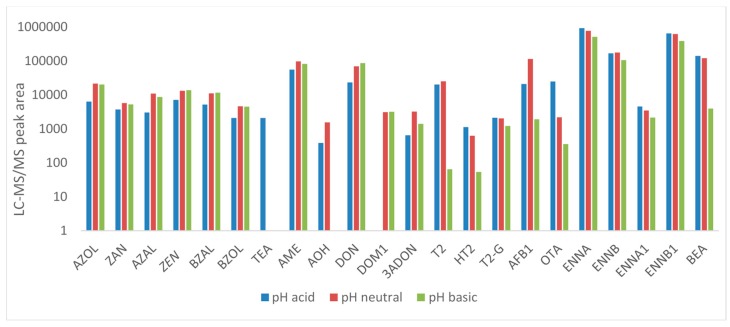
Comparison of the chromatographic peak areas of the different mycotoxins after extraction with ethyl acetate (spiked at 10 ng/mL) from pig urine at three different pH levels: acid (pH 2), neutral (pH 8), and basic (pH 10) (*n* = 2 per protocol).

Since methanol and ACN are mixable with urine, it was not possible to use the same method for urine as for plasma. The use of immunoaffinity columns is avoided due to the high cost. Dilute and shoot methods were eliminated to avoid matrix effects. Therefore, LLE was used to extract the mycotoxins from urine. The main parameters affecting the extraction of all components were optimized: type of extraction solvent, pH, solvent volume and extraction time. During initial experiments, ethyl acetate was evaluated as extraction solvent at neutral pH as in literature extraction of mycotoxins from human urine with this solvent can be found [5,28]. Next, the pH of extraction was optimized using urine spiked at analyte concentrations of 10 ng/mL (*n* = 2 per protocol): acidic (pH 2), neutral (pH 8) and basic (pH 10) extraction conditions were evaluated. Most components showed good extraction at neutral pH (see Figure 3); only for TeA, HT2 and OTA neutral pH showed insufficient results. For these components, significantly higher peak areas were observed at pH 2. This can be explained by the pKa-values of these components. The pKa of TeA (4.5 ± 1) and OTA (3.2 ± 0.1) [45,46] indicate that these components are weak acids and at pH 2 all these components will be neutral and can thus easily be extracted. The pKa values and chemical formulas of the other toxins can be found in Supplementary Table S12. As a result, it was decided to perform two extraction protocols for pig urine, i.e., one in acidic medium (pH 2, tube 1) and another in neutral medium (pH 7, tube 2). Since the same internal standard was used for TeA as for AME and AOH and these components were also detected in acidic medium, they were also added to the acidic protocol. Finally, the volume of ethyl acetate and the extraction time were also optimized. Different volumes (1.5, 3, 5, and 7.5 mL) and different extraction times (15, 30, 45, and 60 min) were tested. It was found that extraction was optimal using a solvent volume of 3 mL and an extraction time of 15 min (results not shown). The combination of both extracts into one vial was not possible since DOM1 and ADON were no longer recovered.

##### Chicken Excreta

**Figure 4:**
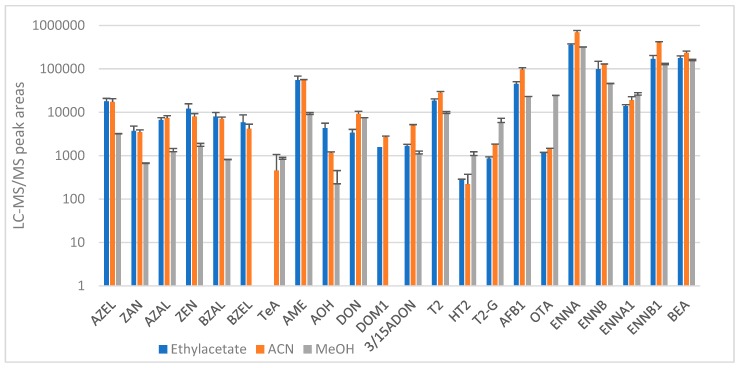
Comparison of the chromatographic peak areas of the different mycotoxins after extraction from broiler chicken excreta (spiked at 10 ng/g) with ethyl acetate, acetonitrile and methanol (*n* = 3 per protocol).

For the excreta of broiler chickens, the same solvents as used for extraction of urine and deproteinization of plasma were evaluated: ACN, MeOH and ethyl acetate. The excreta samples were spiked at an analyte concentration of 10 ng/g (*n* = 3 per protocol). MeOH extraction of broiler chicken excreta did not contain all the metabolites of ZEN. In addition, the peak areas of the measured metabolites of ZEN and DON and AOH/AME were much lower. Next, the chromatograms after extraction with ethyl acetate and ACN were compared. The ethyl acetate extract showed a lower S/N ratio for ZEN and its metabolites. Moreover, the peak areas for the ENNs, AFB1 and DON family were much higher after extraction with ACN compared to ethyl acetate (Figure 4). Therefore, ACN was chosen as optimal extraction solvent. However, to improve the recovery, the influence of the addition of salts (MgSO_4_ and Na_2_SO_4_), acid (HCl), base (NaOH) and water on the extraction recovery was also evaluated. The extraction recovery of TeA and OTA increased by a factor 100 by adding HCl, whereas for all the other components extraction with ACN alone showed the highest recovery. Therefore, it was decided to perform the extraction of chicken excreta twice: with and without HCl (only for TeA and OTA). The final protocol for broiler chicken excreta used 1.5 mL of ACN as an extraction solvent.

##### Pig Feces

The extraction of mycotoxins from pig feces was initially evaluated using the same solvents as for the optimization of chicken excreta: MeOH, ACN, and ethyl acetate. However, these solvents did not give satisfactory results in recovery and the extracts were not sufficiently clean to inject into the instrument. Therefore, other extraction solvents (acetone, diethyl ether) were also evaluated, but the recovery of the mycotoxins was still insufficient. This could be explained by the complexity of the matrix. This challenge was solved by testing different combination of solvents and the combination of the different solvents with SPE columns. This led to a double extraction procedure. The first extraction was a liquid extraction using MeOH/ethyl acetate/formic acid (75/24/1; *v*/*v*) to extract OTA, TeA, AME and AOH. The second protocol to extract the other mycotoxins was a combination of a liquid extraction with acetone and a solid phase extraction with a HybridSPE-phospholipid column. All extraction procedures were tested in triplicate at analyte concentrations of 10 ng/g.

Both feces and excreta extraction required the use of a filtration step using the Millex^®^-LG filter unit (0.2 µm) to obtain samples that were sufficiently clean to inject on the equipment.

For all matrices, the dried extract was reconstituted in 250 µL (or 150 µL for chicken plasma) of MeOH/water (85/15; *v*/*v*). The combination of water and MeOH was crucial to redissolve all mycotoxins with their various physicochemical characteristics.

#### 2.1.2. Optimization of LC-MS/MS and HRMS Parameters

Four different reversed phase columns (Hypersil Gold 50 mm × 2.1 mm, dp: 1.9, Thermo Scientific, Breda, The Netherlands; Zorbax Eclipse C18 50 mm × 2.1 mm, dp: 1.8, Agilent, Sint-Katelijne-Waver, Belgium; Acquity BEH-C18 50 mm × 2.1 mm, dp: 1.7, Waters, Milford, MA, USA; and Acquity HSS-T3 100 mm × 2.1 mm, dp: 1.8, Waters, Milford, MA, USA) were tested to achieve chromatographic separation of the selected mycotoxins. The best separation of all components was obtained on the HSS-T3 column.

The multi-methods were developed with two subsequent analytical runs, i.e., ESI+ and ESI− mode respectively. This was necessary to be able to detect all the mycotoxins with sufficient sensitivity without increasing the run time. Therefore, the mobile phases for each ionization mode were optimized separately. In the literature, the most common mobile phases for mycotoxin detection consist of water and an organic solvent (such as ACN or MeOH). These solvents are often combined with mobile phase modifiers such as volatile acids (formic acid and acetic acid) and ammonium formate or ammonium acetate [34,35]. Different combinations of these solvents and modifiers were evaluated to identify the optimal combination for each ionization mode.

**Figure 5:**
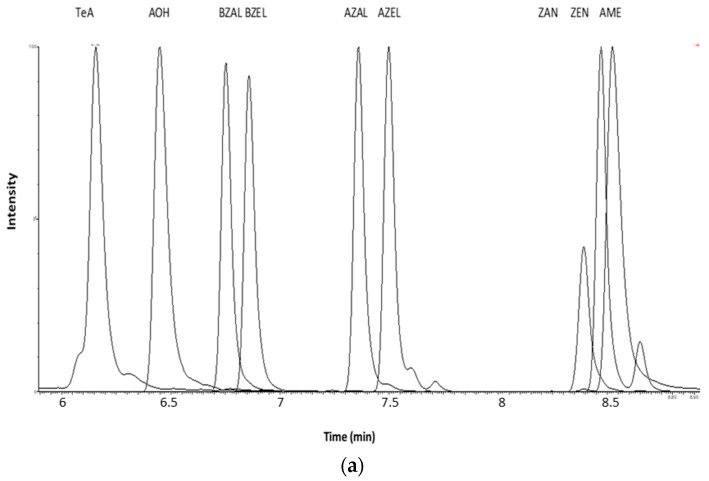

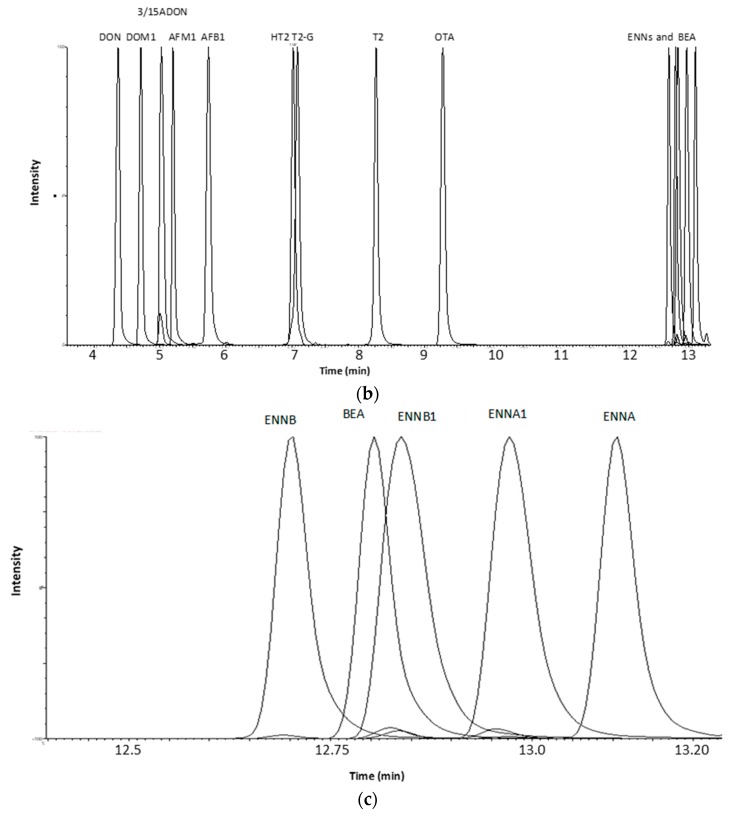
(**a**) LC-MS/MS chromatogram showing the separation of tenuazonic acid (TeA, 6.16 min), alternariol (AOH, 6.45 min), α-zearalanol (AZAL, 7.34 min), α-zearalenol (AZEL, 7.47 min), β-zearalanol (BZAL, 6.73 min), β-zearalenol (BZEL, 6.84 min), zearalanone (ZAN, 8.38 min), zearalenone (ZEN, 8.47 min) and alternariol-monomethyl ether (AME, 8.52 min) at a concentration of 10 ng/mL in broiler chicken plasma; (**b**) LC-MS/MS chromatogram showing the separation of deoxynivalenol (DON, 4.37 min), de-epoxy-deoxynivalenol (DOM1, 4.71 min), 3/15- acetyl-deoxynivalenol (3/15-ADON, 5.02 min), aflatoxin M1 (AFM1, 5.20 min), aflatoxin B1 (AFB1, 5.73), HT2-toxin (HT2, 7.01 min), T2-glucoside (T2G, 7.08 min), T2 toxin (T2, 8.27 min), ochratoxin A(OTA , 9.28 min), beauvericin (BEA) and the enniatins (ENNA, A1, B and B1) in broiler chicken plasma at a concentration of 10 ng/mL; and (**c**) enlargement of (**b**): LC-MS/MS chromatogram showing the separation of BEA and the enniatins (ENNA, A1, B and B1) in broiler chicken plasma at a concentration of 10 ng/mL.

In ESI− mode, ZEN, AZEL, BZEL, AZAL, BZAL, ZAN, TeA, AOH and AME were determined. Baseline separation among ZAN, AZEL, and BZEL as well as between AZAL and BZAL was achieved using water (MP A) and ACN (MP B) as mobile phases [20]. The use of mobile phase with a pH close to neutral led to an impaired peak shape for TeA [47]. The peak shape was optimal when using 1% acetic acid. Therefore, 1% acetic acid in water and 1% acetic acid in ACN were chosen as final mobile phases, since this combination gave satisfactory results for all analytes. Figure 5a shows the chromatographic separation of the mycotoxins in ESI− mode with the optimized parameters as described here.

DON, DOM-1, 3-ADON, 15-ADON, T2, HT2, T2G, OTA, AFB1, AFM1, FB1, ENNA, ENNA1, ENNB, ENNB1 and BEA were determined in ESI+ mode. Taking into account all analytes, the combination of water (MP A) and MeOH (MP B) was most suitable [34]. These mobile phases were further optimized using ammonium formate and formic acid to evaluate the formation of ammonium adducts (M + [NH_4_^+^]). These adducts are generally easier to fragment than sodium adducts, thus enhancing the sensitivity of the method. The final combination of mobile phases was 10 mM ammonium formate and 0.3% formic acid in water (MP A) and in methanol (MP B). Figure 5b,c shows the chromatographic separation of the mycotoxins in ESI+ mode with the optimized parameters as described here.

Since an isotopically labeled IS for each single mycotoxin is too expensive and not commercially available, an IS labeled with [^13^C] or [^15^N] was used for each group of mycotoxins. [^13^C_15_]-deoxynivalenol was used as IS for DON, DOM-1 and 3/15ADON; [^13^C_17_]-Aflatoxin B1 for AFB1 and AFM1; [^13^C_20_]-Ochratoxin A for OTA; [^13^C_34_]-Fumonisin B1 for FB1; [^13^C_6_^15^N]-Tenuazonic acid for TeA, AME and AOH; [^13^C_18_]-Zearalenone for ZEN, AZAL, BZAL, AZEL, BZEL and ZAN; and [^15^N_3_]-Enniatin B for ENNA, ENNA1, ENNB, ENNB1 and BEA. Hence, an optimal correction for matrix effects and losses during sample preparation was obtained, which was confirmed during method validation (see Table 2, Tables S6–S9, and Results Section for accuracy and precision).

Data acquisition on the high-resolution mass spectrometer (HRMS) was done in the positive or negative ESI resolution mode, using the MS^E^ continuum scan function. The results were processed using the Unify version 1.8 software (Milford, MA, USA) to determine the phase I and II metabolite, for which no commercial analytical standards were available.

**Figure 6:**
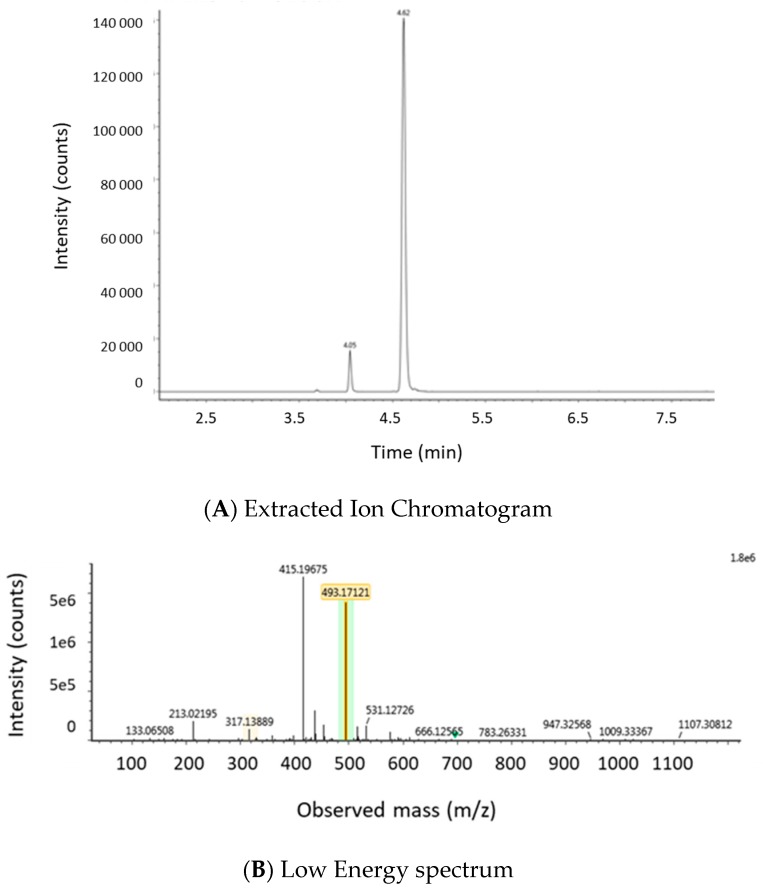

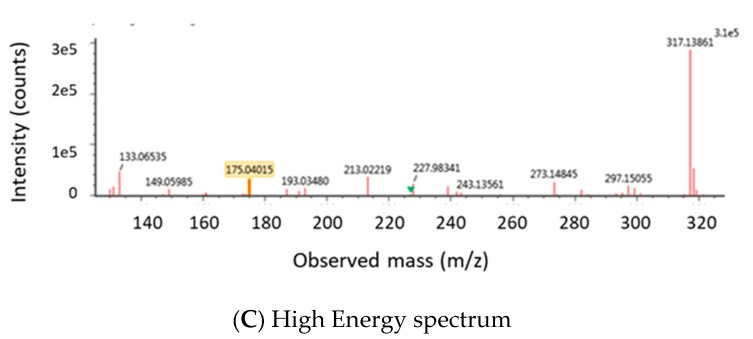
(**A**) LC high-resolution extracted mass chromatogram of a plasma sample that was taken from a pig that received an intra-gastric bolus of zearalenone (ZEN) (3 mg/kg bw); the following mass-to-charge (*m*/*z*) values, corresponding to the theoretical exact mass of the deprotonated molecular ions [M − H]^−^, were extracted from the total ion chromatogram using Unify 1.8 software: ZEN-glucuronide: [*m*/*z* 493.1788]. (**B**) In the low energy MS/MS spectrum, this mass was confirmed as *m*/*z*-value 493.17121 (target mass error = 10 ppm). (**C**) In the high energy MS/MS spectrum, the corresponding product ions at *m*/*z* 317.13877 and 175.04015 (target mass error = 10 ppm) are shown.

Metabolites known in the literature were added to the accurate mass—MSe screening method and additionally a pathway profiling MS^E^ processing method (the chemical formulas and theoretical accurate masses were added) with additional adducts and transformations was made. Peaks were identified based on the found accurate mass in the low energy spectrum and the product ions generated in the high energy spectrum. An additional confirmation criterion was the observed profile of peak areas versus time, seen in the samples obtained during the toxicokinetic study. An example of an extracted ion chromatogram of a glucuronidated metabolite of ZEN and the corresponding low energy and high energy spectra, which was detected in a plasma sample of a pig administered an intra-gastric bolus of ZEN (3 mg/kg bw), is shown in Figure 6.

The Unify version 1.8. software (Waters, Milford, MA, USA) detected two peaks with the exact mass of ZEN-glucuronide [*m*/*z* 494.1788]. However, only the MS/MS spectrum of the second peak (4.68 min) showed the product ions [*m*/*z* 317 and 175] of ZEN-glucuronide as defined in the literature [19]. The first fragment ion (*m*/*z* = 317), corresponds with the loss of glucuronic acid (176 amu) and the second at *m*/*z* = 175 results from the loss of the aglycone from the quasimolecular ion [48]. The data of the first peak can be found in Figure S1.

### 2.2. Method Validation

The most optimal extraction protocol for plasma, urine and feces of pigs and plasma of broiler chickens was not validated for fumonisins due to low recovery. Therefore, it was decided to only validate fumonisin B2 in broiler chicken excreta, hence in this matrix 25 mycotoxins were validated.

The correlation coefficient (r) and the goodness-of-fit (g) are shown in Table 1 as an average ± standard deviation of three curves made across three different analysis days. The linearity results for the other matrices can be found in Tables S2–S5. They ranged for pig plasma from 0.993 to 0.998 (r) and 9.0% to 17.5% (g); for pig urine from 0.995 to 0.999 (r) and 3.5% to 17.0% (g); for pig feces from 0.993 to 0.999 (r) and 7.1% to 18.7% (g); for chicken plasma from 0.994 to 0.999 (r) and 5.9% to 17.7% (g); and for chicken excreta from 0.995 to 0.999 (r) and 5.1% to 16.80% (g). Most of the calibration curves matched a linear calibration model with a 1/x weighing factor, except for the ENNs and BEA. These components show a quadratic 1/x model. Linearity results of each component separately for pig plasma are shown in Table 1.

The LOQ that was aimed for during method development was 1 ng/mL or ng/g. This could be obtained for the majority of the components in the different matrices, with the following exceptions: DOM1 (4 ng/mL) and T2G (2 ng/mL) in pig urine; T2G (2 and 5 ng/mL) in pig and broiler chicken plasma; and T2G (2 ng/g), HT2 (4 ng/g) and FB2 (10 ng/g) in broiler chicken excreta. In pig feces, the LOQ for ZEN, AZAL, AOH, DON, DOM-1, HT2 and T2G was established at 5 ng/g.

No peaks were detected at the retention time zone of the analytes of interest in the solvent sample that was injected after the highest calibrator sample, thus demonstrating the absence of carry over. Moreover, for none of the components a signal was observed at the elution zone of the analytes of interest in a blank matrix sample. This indicates a good specificity of the method.

The results of the within-day and between-day precision and accuracy met the specifications for all mycotoxins and matrices. The results can be found in Table 2 for pig plasma and Tables S6–S9 for the other matrices.

The results for matrix effects (signal enhancement and suppression) and extraction recovery are shown in the Tables S10 and S11. Most components gave acceptable results (range 60–140%). However, for some components, matrix effects were more pronounced and recovery was rather low. However, for all mycotoxins, an adequate internal standard and matrix-matched calibration curves were used, resulting in validation results for accuracy and precision matching the acceptance criteria.

The validation results for the other matrices can be found in Tables S6–S9.

This resulted in a fully validated quantitative targeted LC-MS/MS method and additionally a qualitative untargeted LC-HRMS method. Both methods together enable not only determining mycotoxins with good sensitivity but also targeting a broad range of mycotoxins and their metabolites, not limited by the commercial availability of standards. This approach makes is possible to determine 24 mycotoxins and their relevant metabolites in easily obtainable biological matrices (plasma, urine and feces) of pigs and broiler chickens. This is the first time that a paper covers such a broad range of matrices and mycotoxins with a simple and practical sample preparation. This leads to a general applicable method that can be applied in, among others, in vivo toxicokinetic studies and screening studies to investigate the exposure of individual animals to mycotoxins, as shown in Section 2.3.

### 2.3. Biological Samples: Toxicokinetic Study

#### 2.3.1. Pigs

**Figure 7:**
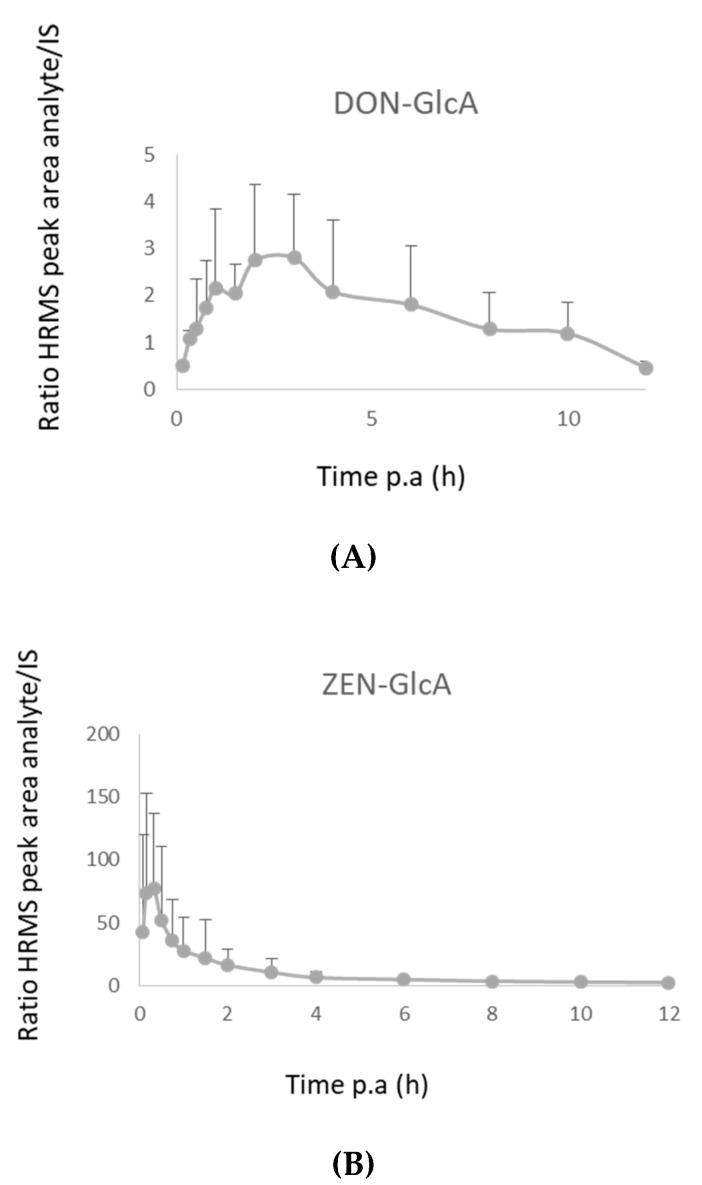
(**A**) HRMS response–time curve of deoxynivalenol-glucuronide (DON-GlcA) in plasma after intra-gastric administration of deoxynivalenol (DON, 36 µg/kg bw) to pigs (*n* = 8). The mean ratio of the HRMS peak areas of DON-GlcA/^13^C_15_-DON + SD is shown. (**B**) HRMS response–time curve of zearalenone-glucuronide (ZEN-GlcA) in plasma after intra-gastric administration of zearalenone (ZEN, 3 mg/kg bw) to pigs (*n* = 8). The mean ratio of the HRMS peak areas of ZEN-GlcA/^13^C_18_-ZEN + SD is shown.

In the pig plasma samples, low concentrations (1–15 ng/mL) of DON and ZEN were found using LC-MS/MS analysis. The plasma concentration–time curves for these components are shown in Figure S2. However, analysis of the samples using the LC-HRMS instrument showed that DON-GlcA and ZEN-GlcA are better biomarkers for exposure as their observed peak areas are much higher than those of the respective parent components DON and ZEN [9,20]. Since no DON-GlcA and ZEN-GlcA standards were available at our laboratory, these components were tentatively identified using the LC-HRMS multi-method. DON-GlcA and ZEN-GlcA plasma response–time curves are shown in Figure 7. The highest response for ZEN-GlcA was achieved at 30 min and for DON-GlcA at 4 h post-administration.

**Figure 8:**
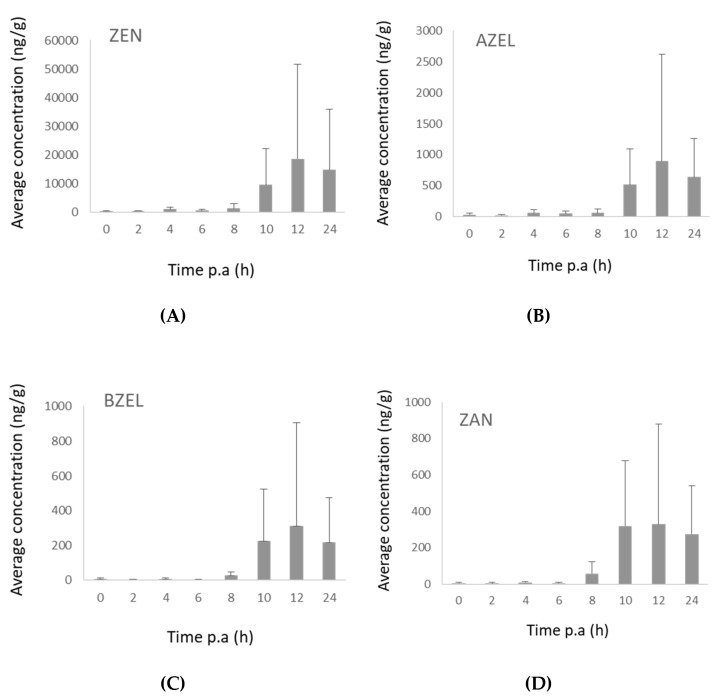
(**A**) Concentration–time curve of zearalenone (ZEN) in feces after intra-gastric administration of ZEN (3 mg/kg bw) to pigs (*n* = 8). The mean + SD is shown. (**B**) Concentration–time curves of α-zearalenol (AZEL) in feces after intra-gastric administration of ZEN (3 mg/kg bw) to pigs (*n* = 8). The mean + SD is shown. (**C**) Concentration–time curve of β-zearalenol (BZEL) in feces after intra-gastric administration of ZEN (3 mg/kg bw) to pigs (*n* = 8). The mean + SD is shown. (**D**) Concentration–time curve zearalanone (ZAN) in feces after intra-gastric administration of ZEN (3 mg/kg bw) to pigs (*n* = 8). The mean + SD is shown.

In pig feces, no traces of DON were observed. This can be explained by the complete absorption and fast elimination of DON in urine, while only 1–3% of the administered dose is reported to be excreted via feces [16]. The concentration–time profiles of ZEN and its phase I metabolites in feces showed maximum levels from the first 12 h after exposure onwards (Figure 8). The highest amounts were excreted during 12–24 h. This is in line with the observations of Binder et al., who found the highest amounts of ZEN and metabolites were excreted during 24–48 h [19].

**Figure 9:**
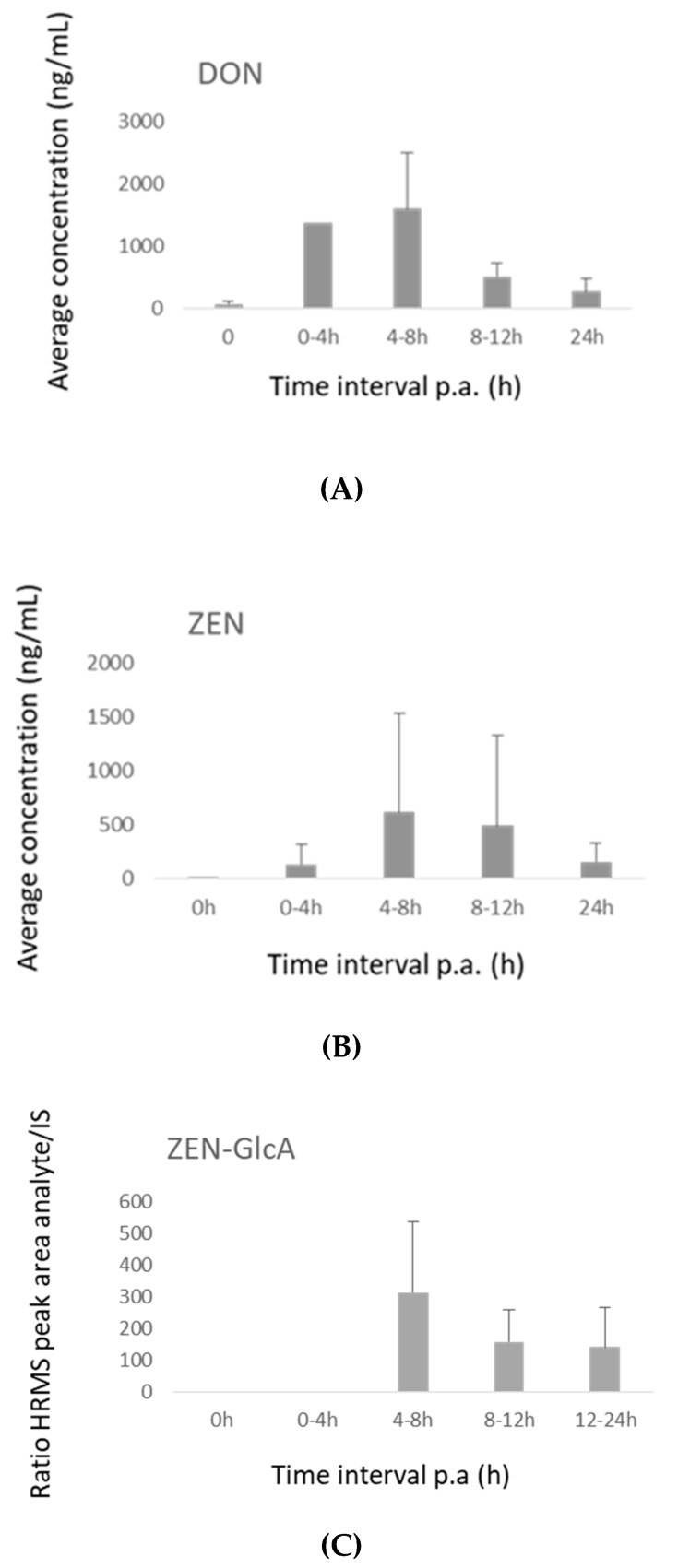
(**A**) Concentration–time curves of deoxynivalenol (DON) in urine after intra-gastric administration of DON (36 µg/kg bw) to pigs (*n* = 8). The mean + SD is shown (**B**) Concentration–time curves of zearalenone (ZEN) in urine after intra-gastric administration of ZEN (3 mg/kg bw) to pigs (*n* = 8). The mean + SD is shown. (**C**) The HRMS response–time curves of zearalenone-glucuronide (ZEN-GlcA) in urine after intra-gastric administration of ZEN (3 mg/kg bw) to pigs (*n* = 8). The mean ratio of the HRMS peak areas of ZEN-GlcA/^13^C_18_-ZEN + SD is shown.

In pig urine, DON, ZEN and ZEN-GlcA were detected. The maximum concentrations were achieved after 4–8 h for ZEN, ZEN-GlcA and DON. Nagl et al. also demonstrated a fast elimination of DON in urine with a maximum concentration in the first 4 h. DON showed to be the most important urinary metabolite [16]. Binder et al. also detected ZEN and ZEN-GlcA in pig urine after oral administration of ZEN, with ZEN-GlcA as the major metabolite. In this study, the response ratio of ZEN-GlcA/IS was also much higher than the area ratio of ZEN/IS, indicating ZEN-GlcA as a major metabolite [19]. The concentration (or HRMS response)–time curves of these molecules are depicted in Figure 9.

In all feces and urine samples, low concentrations (or HRMS response areas) were observed at the time of administration due to the presence of low levels DON and ZEN in the feed. In urine, this concentration was negligible, especially when compared to the concentration after administration. In feces, the effect of the administration was only seen after 10 h. Twelve hours fasting before administration was not enough to eliminate the concentration of mycotoxins in feces after long-term exposure in this study. However, blank samples were obtained in previous studies, as shown in Figure S3.

#### 2.3.2. Broiler Chickens

**Figure 10:**
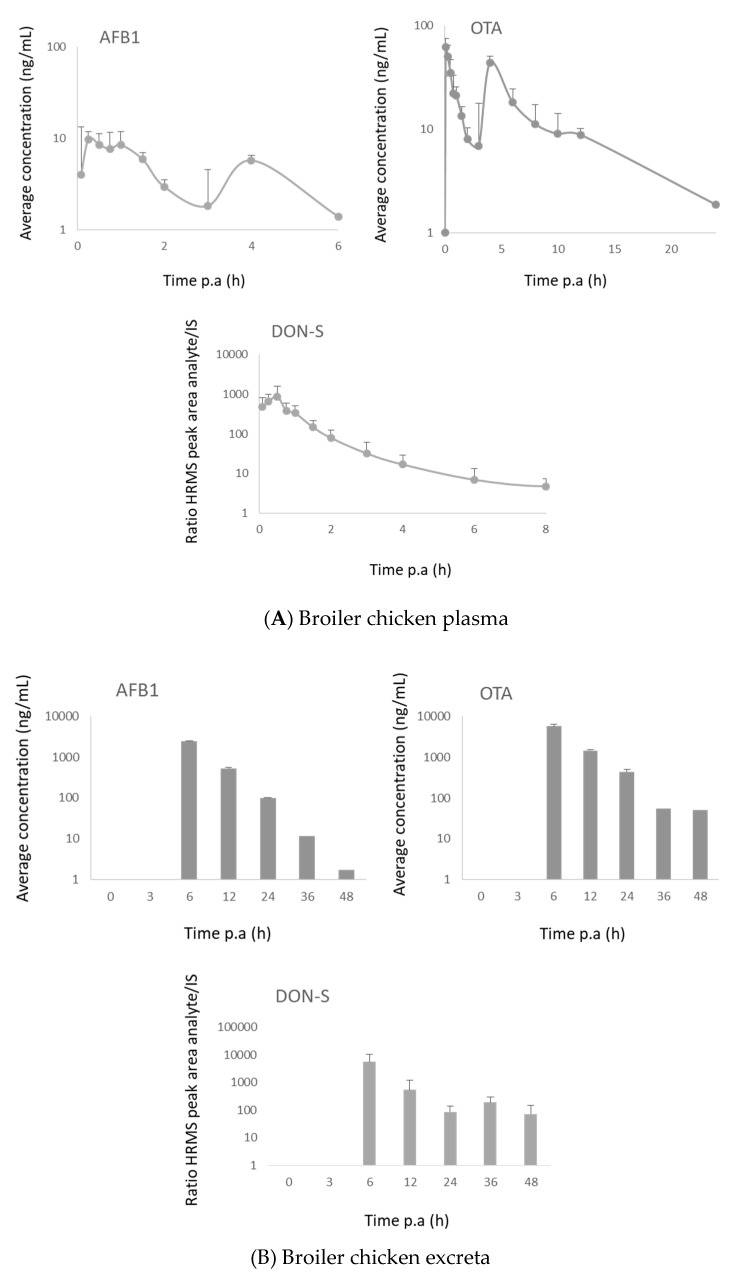
(**A**) Concentration–time curves of aflatoxin B1 (AFB1) and ochratoxin A (OTA), and HRMS response–time curves of deoxynivalenol-sulfate (DON-S) in plasma after PO administration of deoxynivalenol (DON, 0.5 mg/kg bw), AFB1 (2 mg/kg bw) and OTA (0.25 mg/kg bw) to broiler chickens (*n* = 16). The mean + SD is shown for AFB1 and OTA and the mean ratio of the HRMS peak areas of DON-S/^13^C_15_-DON + SD is shown. (**B**) Concentration–time curves of AFB1 and OTA and HRMS response–time curves of DON-sulfate (DON-S) in excreta after PO administration of DON (0.5 mg/kg bw), AFB1 (2 mg/kg bw) and OTA (0.25 mg/kg bw) to broiler chickens (*n* = 16). The mean + SD is shown for AFB1 and OTA and the mean ratio of the HRMS peak areas of DON-S/^13^C_15_-DON + SD is shown.

AFB1 and OTA were detected in plasma, as well as in excreta samples. No other relevant metabolites were found by LC-HRMS. The concentration (response)–time curves are shown in Figure 10. OTA and AFB1 showed a second peak in the plasma concentration–time curve around 4 h p.a. This can be attributed to enterohepatic recirculation, which has previously been described for OTA [49,50]. The maximum concentration measured for AFB1 was 8.4 ng/mL and for OTA was 50 ng/mL, for both toxins observed after 15 min. After administration of DON, only DON-sulfate was found in plasma and excreta due to the high conversion rate of DON to DON-sulfate in broiler chickens, confirming previous literature reports [9,16]. The maximum response was achieved after 30 min.

### 2.4. Screening Study

**Figure 11:**
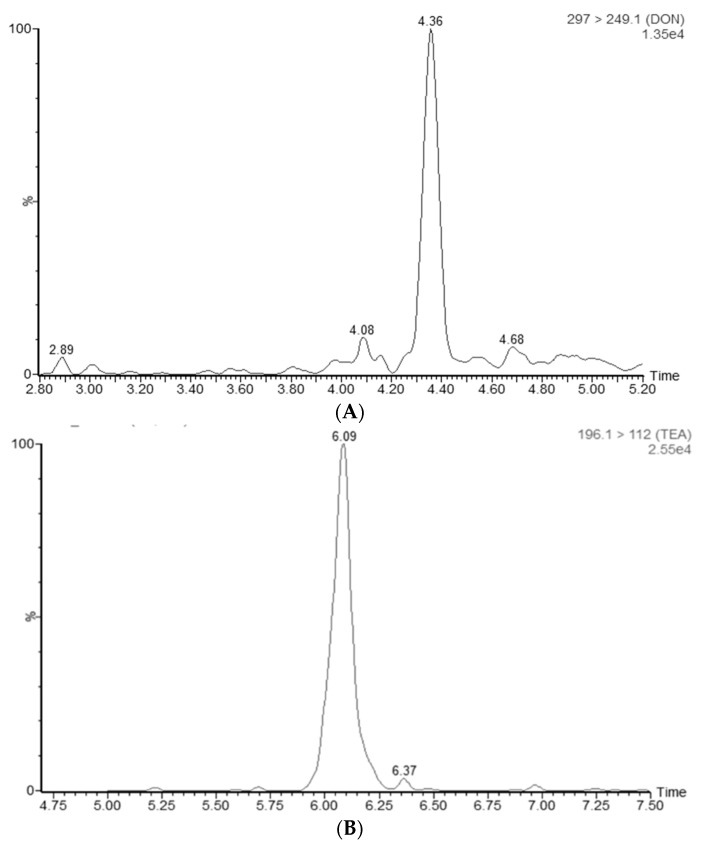

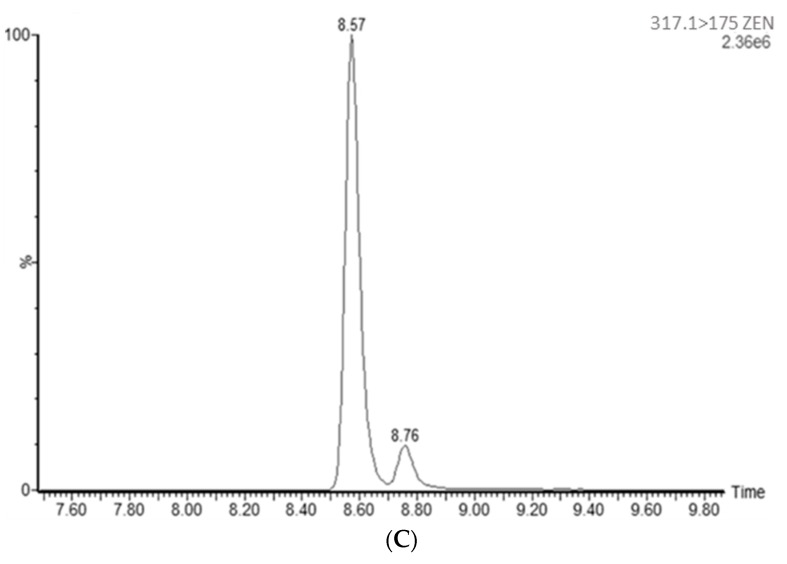
The extracted ion chromatograms showing the presence of: deoxynivalenol (DON) (**A**); tenuazonic acid (TeA) (**B**); and zearalenone (ZEN) (**C**) in pig plasma samples obtained from a farm in Belgium.

Plasma samples from pigs and broiler chickens were obtained from the field and analyzed with the presented method for the presence of mycotoxins in plasma. Two interesting samples are highlighted. The first sample is a pig plasma sample from Belgium. This sample contained ZEN, TeA and DON with respective concentrations of 1, 1.9 and 8.6 ng/mL. The chromatograms are shown in Figure 11.

**Figure 12:**
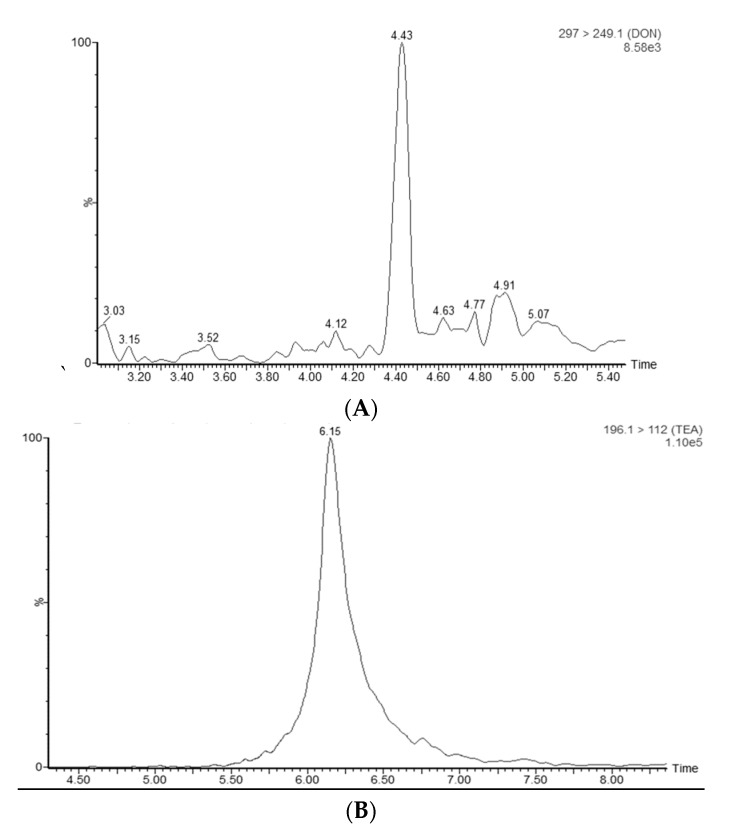
The extraction ion chromatograms showing the presence of: deoxynivalenol (DON) (**A**); and tenuazonic acid (TeA) (**B**) in broiler chicken plasma samples obtained from a farm in Lithuania.

The second sample is a broiler chicken plasma sample from Lithuania. This sample contained TeA and DON with respective concentrations of 1,016 and 70,617 ng/mL. The chromatograms are shown in Figure 12.

## 3. Conclusions

This paper describes a fully validated quantitative targeted LC/MS-MS method, and a qualitative untargeted LC-HRMS approach to determine mycotoxins and their relevant metabolites in easily obtainable biological matrices of pigs and broiler chickens. The methods were applied to plasma, urine, feces and/or excreta samples that were obtained during in vivo toxicokinetic studies with DON and ZEN in pigs, and with DON, AFB1 and OTA in broiler chickens and during a pilot field screening study to monitor exposure to mycotoxins. These results show the successful applicability of the multi-method to pig and broiler chicken samples, providing a proof-of-concept of the developed methods.

## 4. Materials and Methods

### 4.1. Chemicals, Products and Reagents

The analytical standards of ZEN, T2, HT2 toxin (HT2), OTA, AFB1, aflatoxin M1 (AFM1), FB2, AOH, alternariol monomethyl ether (AME), TeA, DON, 3ADON, 15ADON, enniatin A (ENNA), enniatin A1 (ENNA1), enniatin B1 (ENNB1), enniatin B (ENNB) and BEA were obtained from Fermentek (Jerusalem, Israel). Zearalanone (ZAN), α-zearalenol (AZEL), β-zearalenol (BZEL), α-zearalanol (AZAL) and β-zearalanol (BZAL) were purchased from Sigma-Aldrich (Bornem, Belgium). De-epoxy-deoxynivalenol (DOM-1) was obtained from Biopure (Tulln, Austria). T-2 toxin-3α-glucoside (T2G) was synthesized by the U.S. Department of Agriculture (USDA) as described [51,52]. Internal standards (IS) of ^13^C_15_-DON, ^13^C_24_-T2, ^13^C_18_-ZEN, ^13^C_20_-OTA, ^13^C_34_-FB1 and ^13^C_17_-AFB1 were purchased from Biopure. The internal standard ^13^C_6_^15^N-TeA was synthesized according to the method of Asam et al. [53], and ^15^N_3_-ENN B was synthesized according to the method of Hu and Rychlik [54]. All standards were stored according to the recommendations of the supplier. Water, methanol (MeOH), acetonitrile (ACN), ammonium formate, glacial acetic acid and formic acid for the preparation of mobile phases were of LC-MS grade and were obtained from Biosolve (Valkenswaard, The Netherlands). Acetone, ammonium formate, formic acid and ethyl acetate were of analytical grade and were purchased from VWR (Leuven, Belgium). Millex^®^-LG filter units (0.2 µm), sodium hydroxide (NaOH) pellets and hydrochloric acid (HCl) 37% fuming solution were obtained from Merck (Overijse, Belgium). Ostro^®^-96 well plates were obtained from Waters (Milford, MA, USA). HybridSPE^®^-phospholipid 30 mg/1 mL SPE tubes were purchased from Sigma-Aldrich. Merck Alcalit pH indicator paper pH 0–14 was obtained from Novolab (Geraardsbergen, Belgium).

### 4.2. Preparation of Standard Solutions

Standard stock solutions (SS) for ZEN, AZAL, BZAL, AZEL, BZEL, ZAN, DON, T2, T2-G, HT2, AFB1, AFM1, AOH, AME, ENNs, BEA and FB1 were prepared in ACN at 100 µg/mL. Standard SS for OTA was prepared in ACN at 10 µg/mL. The standard SS of TeA was prepared in methanol at 100 µg/mL. Following standards were purchased as solutions: 3ADON (100 µg/mL in ACN), 15ADON (100 µg/mL in ACN) and DOM-1 (50 µg/mL in ACN). A standard SS of 10 µg/mL in ACN was prepared for DOM-1. All internal standards (IS) were obtained as solutions: ^13^C_15_-DON (25 µg/mL in ACN), ^13^C_24_-T2 (25 µg/mL in ACN), ^13^C_18_-ZEN (25 µg/mL in ACN), ^13^C_20_-OTA (10 µg/mL in ACN), ^13^C_34_-FB1 (25 µg/mL in ACN/water) and ^13^C_17_-AFB1 (0.5 µg/mL in ACN). Standard SS of the synthesized internal standards were prepared at a concentration of 100 µg/mL in MeOH for ^13^C_6_^15^N-TeA and 10 µg/mL in ACN for ^15^N_3_-ENN B. The SS were stored at ≤−15 °C.

A combined working solution of all analytical standards (WS_mix_, without IS) at a concentration of 1 µg/mL was prepared by transferring 10 µL of the stock solutions with a concentration of 100 µg/mL and 100 µL of a 10 µg/mL solution of DOM-1 and OTA, followed by further dilution with ACN up to a total volume of 1 mL. Serial dilutions of the WS_mix_ were prepared, yielding concentrations of 100 ng/mL and 10 ng/mL. Individual working solutions of 1 µg/mL were made for all IS, except for ^13^C_17_-AFB1 (100 ng/mL) and ^15^N_3_-ENN B (100 ng/mL). Next, a combined working solution of all IS (WS_mix_IS_) was prepared with a final concentration of 100 ng/mL for all components, except ^13^C_17_-AFB1 (10 ng/mL) and ^15^N_3_-ENN B (10 ng/mL). All working solutions were stored at ≤−15 °C.

### 4.3. Biological Samples

A toxicokinetic study was performed to demonstrate the applicability of the developed method. Incurred plasma samples were obtained from 8 hybrid pigs (6 weeks of age, 9.94 ± 1.24 kg, ♂/♀ 4/4), dosed with a single oral (intragastric) bolus of DON (36 µg/kg bodyweight (bw)) and ZEN (3 mg/kg bw) after a fasting period of 12 h, and from 16 12 h-fasted broiler chickens (Ross 308, 3 weeks of age, 1.05 ± 0.11 kg, ♂/♀ 5/11) administered AFB1 (2 mg/kg bw), DON (0.5 mg/kg bw) and OTA (0.25 mg/kg bw). All mycotoxin doses were administered as a single oral bolus (acute exposure, by gavage), and corresponded with doses previously used in toxicokinetic studies and studies to test the efficacy of mycotoxin detoxifiers. For DON, this dose was in agreement with the EU legislation in feed. The maximum guidance level in pig feed is set at 0.9 mg/kg DON [3]. Pigs of this age category consume on average 40 g feed/kg bw/day. This resulted in the administration of 36 μg DON/kg bw as described in [8]. For broiler chickens, the EU regulations set the maximum guidance level at 5 mg/kg feed [3]. Broilers (±1 kg bw) consume on average 100 g feed/kg bw/day. This resulted in the administration of 0.5 mg DON/kg bw as described in [8].

For ZEN in pigs and AFB1 and OTA in broiler chickens, the administered doses were higher than set by the EU legislation. These doses corresponded to the doses previously administered in toxicokinetic studies and studies to determine the efficacy of mycotoxin detoxifiers [50]. These higher doses were necessary to obtain sufficiently high plasma concentrations to evaluate the toxicokinetic parameters of the toxins, as well as to demonstrate the efficacy of mycotoxin detoxifiers. Although the doses were higher than the guidance levels, they did not evoke clinical toxicity after this single administration. This was the case in the previously mentioned studies [50], as well as in the present study. The most important read-out of this single oral bolus dosing in animals is the area under the plasma concentration–time curve (AUC), which has to be high enough to be able to demonstrate a statistical significant reduction in AUC when combined with the detoxifier. As the goal here was to evaluate the use of the analytical methods in this type of studies, the same doses were used.

Blood was sampled before administration (0 min) and at 5, 10, 20, 30, 45, 60, 90, and 120 min, and 3, 4, 6, 8, 10, 12 and 24 h after administration of mycotoxins. Blood was collected via the vena jugularis in EDTA tubes using a Venoject^®^ system and centrifuged (2851× *g*, 10 min, 4 °C) to obtain plasma, which was stored at ≤−15 °C until analysis. Urine was collected from male pigs using pediatric urine collection bags as described by Gasthuys et al.[55]. Sampling was done at 4 intervals: 0–4 h, 4–8 h, 8–12 h and 12–24 h. Feces were collected every 2 h by rectal stimulation of the pigs. Excreta were collected every 2 h by placing the broiler chickens in separate boxes for sampling. The animal trial was approved by the ethical committee of the Faculty of Veterinary Medicine and the Faculty of Bioscience Engineering of Ghent University (EC2017/05) on 30 March 2017.

A multi-mycotoxin LC-MS/MS analysis of the pig and chicken feed (Primoris, Zwijnaarde, Belgium) showed only low amounts of DON (respectively, 139 and 140 µg/kg) and ZEN (respectively, 12 and 20 µg/kg). The feed was conform the EU legislation since these amounts are below the guidance values of the European commission [3]. Blank plasma, urine and feces samples were obtained from pigs and broiler chickens on the mycotoxin control diet. The blank samples were used for the preparation of matrix-matched calibration curves and quality control samples.

Besides the toxicokinetic studies, a preliminary screening study (*n* = 1 farm per animal species) was performed to monitor the exposure of pigs and broiler chickens to mycotoxins and to demonstrate the applicability of the developed method in the field. Therefore, farms with problems that might be related to mycotoxins (e.g., postpartum problems, tail necrosis and refused feed intake) and where mycotoxins were found in feed were selected. Blood of 10 pigs (5 sows and 5 piglets) and 10 broiler chickens (2 weeks of age) was collected around 30 min after feeding. The blood was collected in EDTA tubes and centrifuged (2851× *g*, 10 min, 4 °C) to obtain plasma. The animal trial was also approved by the ethical committee of the Faculty of Veterinary Medicine and the Faculty of Bioscience Engineering of Ghent University (EC2017/115) on 30 March 2017.

### 4.4. Sample Pre-Treatment

#### 4.4.1. Pig Plasma

To 250 µL of plasma, 20 µL of a 100 ng/mL WS_mix_IS_ were added, vortex mixed and set for equilibration at room temperature for 5 min. Next, 750 µL of ACN with 0.1% formic acid of analytical grade were added, followed by vortex mixing (10 s) and centrifugation (8,517× *g*, 10 min, 4 °C). The total supernatant was collected and dried under a nitrogen (N_2_) stream at 40 ± 5 °C. The dried supernatant was reconstituted in 250 µL of methanol/water (85/15; *v*/*v*), followed by vortex mixing. The reconstituted sample was transferred into an autosampler vial and an aliquot (5 µL) was injected onto the LC-MS/MS and LC-HRMS instrument.

#### 4.4.2. Pig Urine

To two tubes (tube 1 and 2), each containing 500 µL of urine, were added 20 µL of a 100 ng/mL WS_mix_IS_, followed by vortex mixing and equilibration at room temperature for 5 min. The pH was determined by means of pH test strips and was adjusted to pH 8 in tube 1 and to pH 2 in tube 2, using a 0.1 M NaOH solution and a 1 M HCl solution, respectively. This pH adjustment was necessary to allow extraction of the different mycotoxins. At pH 8 most mycotoxins were extracted except for TeA, AOH, AME and OTA that extracted best at pH 2.

Thereafter, 3 mL of ethyl acetate were added to each tube, followed by vortex mixing for 10 s and rotating during 15 min on a horizontal roller shaker (Staffordshire, UK). Finally, the tubes were centrifuged for 10 min at 3724× *g* and 4 °C. The organic phase of each tube was transferred to separate polypropylene tube and evaporated to dryness using a gentle N_2_-stream at 40 ± 5 °C. The dried extracts of tube 1 and 2 were reconstituted in 250 µL of MeOH/water (85/15; *v*/*v*) and vortex mixed. The redissolved extracts of tube 1 and 2 were transferred to a separate autosampler vial and an aliquot (5 µL) was injected onto the analytical instruments.

#### 4.4.3. Pig Feces

Feces samples were first freeze dried for 48 h to eliminate variation due to different moisture contents. Then, 20 µL of a 100 ng/mL WS_mix_IS_ were added to two tubes (tube 1 and 2), each containing 250 mg of freeze-dried feces. The samples were vortex mixed during 10 s and equilibrated at room temperature for 5 min. The extraction of TeA, OTA, AME and AOH was performed using tube 2, with 5 mL of methanol/ethyl acetate/formic acid of analytical grade (75/24/1; *v*/*v*), whereas the other mycotoxins were extracted using tube 1 with 5 mL of acetone. Both tubes were shaken for 40 min on an in-house made vertical rotator (75 rpm), followed by centrifugation (3724× *g*, 10 min, 4 °C). The supernatant of tube 1 was transferred onto a HybridSPE-phospholipid cartridge. The eluate of tube 1 and the supernatant of tube 2 were evaporated until dryness using a gentle N_2_-stream at 40 ± 5 °C. The dried extracts were reconstituted in 250 µL of MeOH/water (85/15; *v*/*v*) and filtered through a Millex^®^-LG 0.2 µm filter. The redissoved extracts of tube 1 and 2 were transferred to a separate vial and an aliquot (5 µL) was injected onto the LC-MS/MS and the LC-HRMS instrument.

#### 4.4.4. Broiler Chicken Plasma

First, 150 µL of chicken plasma was brought in a well of an Ostro^®^ 96-well plate. Next, 15 µL of a 100 ng/mL WS_mix_IS_ were added, followed by a gentle up and down pipetting for mixing and equilibration for 5 min at room temperature. Next, 450 µL of ACN containing 0.1% formic acid of analytical were added. After gentle mixing, the Ostro^®^ 96-well plate was placed under vacuum (67.7 kPa) to allow the sample to pass through the plate. The eluate of each sample was transferred to a polypropylene tube, dried under a gentle N_2_-stream at 40 ± 5 °C and reconstituted in 150 µL of MeOH/water (85/15; *v*/*v*). An aliquot of 5 µL was injected onto the analytical instruments.

#### 4.4.5. Broiler Chicken Excreta

Excreta samples were first freeze dried for 48 h to eliminate variation due to different moisture contents. The extraction of TEA, AME, OTA and AOH was performed in acidic medium (tube 1), whereas the other mycotoxins were extracted without adjustment of the pH (tube 2). Hence, 250 mg of freeze-dried chicken excreta were added to tube 1 and 2, followed by the addition of 20 µL of a 100 ng/mL WS_mix_IS_ and equilibration for 5 min at room temperature. Next 1.5 mL of ACN was added to both tubes, followed by adding 250 µL of a 1 M HCl solution to tube 1. The pH of the excreta in tube 2 was not adjusted. The two tubes were vortex mixed and shaken for 15 min on a vertical rotator, followed by centrifugation (3724× *g*, 10 min, 4 °C). The supernatants were transferred to separate polypropylene tubes and dried under a N_2_-stream at 40 ± 5 °C. The dried extracts were reconstituted in 250 µL of MeoH/water (85/15; *v*/*v*) and filtered through a Millex^®^-LG 0.2 µm filter. Each sample was transferred to a separate vial. An aliquot of 5 µL was injected onto the LC-MS/MS and LC-HRMS instruments.

### 4.5. Chromatography

The chromatographic systems consisted of an Acquity H-Class ultra-performance liquid chromatograph (UPLC) coupled to a Xevo^®^ TQ-S mass spectrometer and an Acquity I-Class UPLC coupled to a Synapt^®^ G2-Si high definition mass spectrometer (HDMS), all from Waters. Chromatographic separation was achieved on an Acquity HSS T3 column (100 mm × 2.1 mm i.d., dp: 1.8 μm) and a VanGuard pre-column of the same type (5 mm × 2.1 mm i.d., dp: 1.8 μm), both from Waters. The temperatures of the column oven and autosampler tray were set at 45 °C and 8 °C, respectively.

The chromatographic conditions were optimized for the different ionization modes, i.e., positive and negative electro-spray ionization (ESI). The optimal chromatographic conditions for the ESI positive mode were obtained with the mobile phases (MP) containing 10 mM ammonium formate, 0.3% formic acid in water (MP A), and 10 mM ammonium formate, 0.3% formic acid in methanol (MP B), all of LC-MS grade. In ESI negative mode, the most suitable mobile phases consisted of 1% acetic acid in water (MP C) and 1% acetic acid in acetonitrile (MP D), all of LC-MS grade. A gradient elution program was run for each ionization mode separately. For ESI positive: 0–1.5 min, 95% A, 5% B; 1.5–3 min, linear gradient to 40% A; 3–5 min, 40% A, 60% B; 5.0–10 min, linear gradient to 20% A; 10–10.50 min, linear gradient to 1% A; 10.50–13.0 min, 1% A, 99% B; 13–14 min, linear gradient 95% A; 14.0–16.0 min, 95% A, 5% B. For ESI negative: 0–1.5 min, 95% C, 5% D; 1.5–3 min, linear gradient to 60% C; 3.0–4.0 min, 60% C, 40% D; 4.0–7.0 min, linear gradient to 40% C; 7.0–9.0 min, 40% C, 60% D; 9.0–9.5 min, linear gradient 95% C; 9.5–12.0 min, 95% C, 5% D. The flow rate was set at 300 μL/min. The chromatographic parameters were the same for both the LC-MS/MS and LC-HRMS instruments.

### 4.6. Mass Spectrometry

Instrument parameters were optimized by syringe infusion of working solutions of 10 µg/mL of each compound (flow rate 10 µL/min).

#### 4.6.1. LC-MS/MS

The settings on the Xevo^®^ TQ-S mass spectrometer were as follows: desolvation gas flow rate: 800 L/h; desolvation temperature: 600 °C; cone gas flow rate: 150 L/h; source temperature: 150 °C. The capillary voltage was optimized at 3.2 kV for ESI positive and 3.0 kV for ESI negative mode, respectively. Dwell times of 25 and 10 ms/transition were selected for each component separately. The Xevo^®^ TQ-S mass spectrometer was operated in the selected reaction monitoring (SRM) mode. For every compound, the two most intense product ions were selected for quantification and qualification, respectively. In Table 3; Table 4, an overview is given of the compound specific MS/MS parameters at the selected ionization mode (ESI negative and ESI positive, respectively).

#### 4.6.2. LC-HRMS

Following instrument parameters were selected: desolvation gas flow: 800 L/h; desolvation temperature: 600 °C; cone gas flow: 50 L/h; source temperature: 150 °C. The capillary voltage was 3.2 kV for ESI positive and and 3.0 kV for ESI negative. The HRMS acquisition was performed in resolution mode using the MS^E^ continuum scan function. The MS^E^ data acquisition was optimal for use in the Unify 1.8. software (Waters, Milford, MA, USA). The settings were as follows: low mass, 50 dalton (Da); high mass, 1200 Da; scan time, 0.1 s; data format, continuum. The lock mass solution consisted of leucine encephalin (200 pg/µL). The lock spray capillary voltage was 2.8 kV for positive and 2.15 kV for the negative ionisation mode. The additional lock spray settings were as follows: scan time, 0.1 s; interval, 30 s; scans to average, 3; mass window, 0.5 Da. The lock spray was acquired during HRMS acquisition, but not corrected. The lock spray correction (*m*/*z* 556.276575; *m*/*z* 554.26202) and data processing was performed using Unify 1.8 software (Waters, Milford, MA, USA). Identification of analytes for which analytical standards were available was based on retention time (target T_R_ tolerance: 0.1 min) and mass (target mass tolerance: 10 ppm). Identification of phase I and II metabolites for which no analytical standards existed was based on the found exact mass (target mass tolerance: 10 ppm).

For every mycotoxin and some of their phase I and II metabolites, the accurate masses were defined in the Unify 1.8 processing method, as shown in Table S1. The search for phase I and II metabolites was performed using a pathway profiling approach. Additional adducts (e.g., Na^+^, NH_4_^+^, CH_3_COO^−^, HCOO^−^) and the following transformations were added to the method: glucuronidation, sulfation, oxidation, glutathione conjugations, glucosylations. After detection of a peak based on the accurate mass by Unify 1.8, the given MS/MS spectrum was inspected to confirm the proposed structure based on the present product ions.

### 4.7. Method Validation

The LC-MS/MS method was validated according to a protocol previously described by De Baere et al. [35], using spiked blank plasma, urine and feces samples obtained from healthy, untreated animals. The validation requirements are in compliance with the recommendations and guidelines defined by the European and international Community [56,57,58]. Following parameters were evaluated: linearity, within-day and between-day precision and accuracy, limit of quantification (LOQ), carry over, specificity, extraction recovery and matrix effects.

#### 4.7.1. Linearity

Linearity was assessed by preparing three matrix-matched calibration curves over a concentration range of 1–200 ng/mL or 1–200 ng/g. Ten concentrations were included: 0, 1, 2, 4, 5, 10, 30, 50, 100 and 200 ng/mL or ng/g. The correlation coefficients (r) and the goodness-of-fit coefficients (g) were calculated and acceptance criteria were set, respectively, at ≥0.99 and ≤20% [59]. 

#### 4.7.2. Precision and Accuracy

Within-day precision and accuracy were evaluated by analyzing six blank samples spiked at low (LOQ), medium (10 ng/mL or ng/g) and high (100 ng/mL or ng/g) concentration levels. Between-day precision and accuracy were determined by analyzing in threefold three quality control samples spiked at low (LOQ), medium (10 ng/mL or ng/g) and high (100 ng/mL or ng/g) concentration level on three different days. The acceptance criteria for within-day and between-day accuracy were as follows: −50% to +20%, −30% to +10% and −20 to +10% for concentrations of ≤1 ng/mL or ng/g, 1–10 ng/mL or ng/g and ≥10 ng/mL or ng/g, respectively. For the within-day precision, the relative standard deviation (RSD%) had to be lower than the maximum relative standard deviation (RSDmax), which was <25%, and <15% for concentrations ≥1 to <10 ng/mL or ng/g and ≥10 to <100 ng/mL or ng/g, respectively [58]. For the between-day precision, the RSD% had to be lower than the RSDmax, which was defined by the Horwitz equation: RSDmax = 2^(1–0.5 log Concentration(g/mL))^. The RSDmax was 22.6%, 32% and 45% for the respective concentrations of 100 ng/mL, 10 ng/mL and 1 ng/mL [56,57].

#### 4.7.3. Limit of Quantification (LOQ)

The LOQ was the lowest concentration of the analyte for which the method was validated with an acceptable accuracy and precision according to the guidelines described above. The LOQ was also the lowest concentration of the calibration curves. The LOQ was determined by analyzing different concentrations spiked in six-fold on the same day.

#### 4.7.4. Carry Over

Carry over was assessed by analyzing a mixture of MeOH/water (85/15; *v*/*v*) directly after the highest calibrator (200 ng/mL or ng/g). The concentration of mycotoxins in this mixture had to be below a signal-to-noise ratio of 3/1.

#### 4.7.5. Specificity

The specificity of the method was evaluated with respect to interference of endogenous components. Hence, a blank sample was analyzed. The signal of the eventual interference at the elution zone of the analytes of interest should be below the signal-to-noise ratio of 3/1.

#### 4.7.6. Extraction Recovery and Matrix Effects

The extraction recovery and matrix effects of the method were calculated according to the method of Matuszewski et al. [60] Therefore, three types of samples were prepared. The first samples were matrix-matched and prepared by spiking blank samples before extraction (=Spiked). The second samples consisted of matrix-matched blank samples that were spiked after extraction (=SpikedExtract). The third samples were prepared using standard solutions (=Standard). All samples were spiked at 10 ng/mL or 10 ng/g and were made in triplicate. The peak areas of these samples were compared to calculate the recovery of the extraction step (RE) and the signal suppression/enhancement (SSE) due to matrix effects.

SSE = 100 × (Area SpikedExtract/Area Standard) RE = 100 × (Area Spiked/Area SpikedExtract)

## Figures and Tables

**Table 1:** Validation results for linearity (linear range, correlation coefficient (r) and goodness-of-fit coefficient (g)) and limit of quantification (LOQ) of 24 mycotoxins in pig plasma.

Analyte	Linearity (*n* = 3 Different Days)			LOQ (ng/mL)
	Linear Range (ng/mL)	r ± SD	g (%) ± SD	
ZEN	1–200	0.996 ± 0.003	12.9 ± 3.8	1.0
AZEL	1–200	0.995 ± 0.002	15.0 ± 1.2	1.0
AZAL	1–200	0.995± 0.003	15.2 ± 3.6	1.0
BZAL	1–200	0.996 ± 0.001	10.7 ± 2.0	1.0
BZEL	1–200	0.996 ± 0.002	14.9 ± 3.7	1.0
ZAN	1–200	0.997 ± 0.001	16.3 ± 3.1	1.0
TEA	1–200	0.998 ± 0.001	12.0 ± 4.9	1.0
AOH	1–200	0.997 ± 0.002	12.9 ± 5.2	1.0
AME	1–200	0.996± 0.004	13.1± 5.1	1.0
DON	1–200	0.998 ± 0.002	13.9 ± 4.4	1.0
DOM-1	1–200	0.997 ± 0.003	16.9 ± 2.5	1.0
3/15 ADON	1–200	0.998 ± 0.001	9.3 ± 3.1	1.0
T2	1–200	0.998 ± 0.001	9.9 ± 1.6	1.0
HT2	1–100	0.993 ± 0.002	17.5 ± 2.8	1.0
T2G	2–200	0.995 ± 0.003	14.4 ± 1.2	2.0
AFB1	1–200	0.996 ± 0.002	12.7 ± 2.8	1.0
AFM1	1–200	0.997 ± 0.002	13.8 ± 5.6	1.0
OTA	1–200	0.993 ±0.004	9.3 ± 2.9	1.0
ENNA1	1–200	0.998 ± 0.001	9.0 ± 0.9	1.0
ENNA	1–50	0.995 ± 0.003	13.9 ± 3.2	1.0
ENNB	1–100	0.993 ± 0.002	9.3 ± 1.2	1.0
ENNB1	1–100	0.998 ± 0.001	15.9 ± 2.3	1.0
BEA	1–100	0.998 ± 0.000	16.6 ± 4.5	1.0

Note: SD, standard deviation; acceptance criteria: r ≥ 0.990 and g ≤ 20.

**Table 2:** Results of the within-day and between-day precision and accuracy experiments for 24 mycotoxins in pig plasma.

Within-Day Precision and Accuracy (*n* = 6)							Between-Day Precision and Accuracy (*n* = 3 × 3)					
Analyte	Theoretical Concentration LOQ		Theoretical Concentration 10 ng/mL		Theoretical Concentration 100 ng/mL		Theoretical Concentration LOQ		Theoretical Concentration 10 ng/mL		Theoretical Concentration 100 ng/mL	
	Precision	Accuracy	Precision	Accuracy	Precision	Accuracy	Precision	Accuracy	Precision	Accuracy	Precision	Accuracy
	(RSD %)	(%)	(RSD %)	(%)	(RSD %)	(%)	(RSD %)	(%)	(RSD %)	(%)	(RSD %)	(%)
ZEN	12.1	6.5	7.3	1	5.4	−1.5	13.4	2.7	7.5	2.4	5.7	−2.2
AZEL	6.5	19.7	13	2.3	7.4	6.6	35.4	−5.3	10.9	−0.7	11.6	1.5
AZAL	17.4	−14.5	7.7	−1.4	4.4	−0.4	20.9	−3.3	8.2	−5.2	8.1	−4.9
BZAL	5.2	7	3.5	8.2	2.9	8.9	9.3	6.2	3.9	6.6	10.7	4.2
BZEL	13.0	−9.2	5.1	−1.8	6.1	−2.4	15.9	−1.8	8.1	−4.9	6.6	−5.3
ZAN	11.7	−40.5	3.9	8.8	4.6	3.0	21.5	−32.4	3.6	9.8	7.1	0.4
TEA	2.8	19.0	3.2	8.2	3.6	4.5	10.7	18.2	3.4	8.7	5.8	4.8
AOH	17.6	−32.2	3.5	8.6	3.8	−0.4	26.9	−20.8	4.5	9.5	5.3	−3.1
AME	14.1	10.1	7.7	2.4	4.8	−12.3	18.7	−5.3	7.0	4.8	5.9	−11.9
DON	24.9	−8.0	6.9	1.0	5.6	−5.7	22.3	4.4	10.0	2.3	8	−4.6
DOM-1	17.5	−0.4	15.0	−3.3	5.9	−6.9	14.7	−2.0	14	−2.7	7.9	−7.3
3/15 ADON	15.6	7.5	5.2	5.2	6.4	−3.5	16.4	10.9	5.4	6.9	8.6	−3.5
T2	15.4	3.3	1.6	7.7	2.7	8.4	12.7	3.7	1.5	8.1	6.2	5.0
HT2	21.1	−29.8	6.5	−14.0	9.9	5.8	30.5	−21.4	10.0	−11.5	5.7	2.1
T2G	10.8	−3.8	7.8	6.9	7.0	1.3	23.8	−2.6	9.5	4.1	13.8	6.6
AFB1	13.1	−14.3	3.0	3.9	4.9	−2.0	16.4	−16.9	3.6	4.5	6.3	−2.8
AFM1	11.2	−38.8	10.6	−19.5	8.8	−15.6	28.0	−28.5	18.8	−7.1	20.2	−5.4
OTA	7.5	13.4	8.7	−13.3	4.5	−12.3	14.4	2.5	7.2	−12.5	9.2	−8.0
ENN A1	15.7	−11.3	12.6	−3.7	6.7	1.8	14.6	−2.3	10.8	−0.8	9.6	−0.9
ENNA	19.4	−1.0	9.4	−14.1	11.7	−14.7	41.7	−11.2	13.8	−6.8	13.5	−5.6
ENNB	16.7	−0.1	11.8	9.6	2.6	9.4	16.8	−1.4	13.9	−1.3	6.6	4.1
ENNB1	7.6	16.1	3.9	−0.1	3.5	−3.9	31.8	1.6	8.7	5.5	3.2	−3.2
BEA	13.3	−2.9	3.2	6.9	3.2	7.4	29.7	−6.3	2.3	7.2	11.9	8.5

Note: The acceptance criteria: Accuracy, ≤1 ng/mL: −50% to +20%; 1–10 ng/mL: −30% to +10%; ≥10ng/mL: −20% to +10%. Within-day precision: RSD% < RSDmax with RSDmax for ≥1 to <10 ng/mL: <25% and ≥10 to <100 ng/mL: <15%. Between-day precision: the RSD% < RSDmax with RSDmax 22.6%, 32% and 45% for the respective concentrations of 100 ng/mL, 10 ng/mL and 1 ng/mL, respectively.

**Table 3:** Overview of the compound specific MS/MS parameters for mycotoxins, measured in the ESI negative mode.

Name	Measured Form/Adduct	Precursor Ion (*m*/*z*)	Quantifier Ion (*m*/*z*)	Qualifier Ion (*m*/*z*)	Cone Voltage (V)	Collision Energy (eV) (a-b)	Retention Time (min)
ZEN	[M − H]^−^	317.1	175.0	130.8	15	25-30	8.50
ZAN	[M − H]^−^	319.1	275.0	205.0	20	20-22	8.41
BZEL	[M − H]^−^	319.2	275.0	301.0	20	20-22	6.87
BZAL	[M − H]^−^	321.2	277.2	303.3	30	23-20	6.75
AZEL	[M − H]^−^	319.2	275.0	301.0	20	20-22	7.51
AZAL	[M − H]^−^	321.2	277.2	303.3	30	23-20	7.36
TeA	[M − H]^−^	196.1	112.0	139.0	55	23-23	6.06
AOH	[M − H]^−^	256.8	213.0	185.2	20	28-28	6.33
AME	[M − H]^−^	271.1	256.0	228.0	48	24-30	8.38
[^13^C_18_]-zearalenone	[M − H]^−^	335.3	185.1	169.1	15	28-40	8.50
[^13^C_6_^15^N]-tenuazonic acid	[M − H]^−^	202.9	113.1	141.9	40	23-20	6.06

Note: *m*/*z* = mass-to-charge ratio; (a-b): collision energy for the quantifier (a) and qualifier ion (b), respectively.

**Table 4:** Overview of the compound specific MS/MS parameters for mycotoxins, measured in the ESI positive mode.

Name	Measured Form/Adduct	Precursor Ion (*m*/*z*)	Quantifier Ion (*m*/*z*)	Qualifier Ion (*m*/*z*)	Cone Voltage (V)	Collision Energy (eV) (a-b)	Retention Time (min)
DON	[M + H]^+^	297.0	249.1	203.4	20	9-14	4.36
DOM1	[M + H]^+^	281.0	215.0	233.0	30	12-12	4.70
3/15-ADON	[M + H]^+^	339.2	213.1	230.9	25	12-8	5.02
T2	[M + NH_4_]^+^	484.0	215.2	304.8	26	18-15	8.24
HT2	[M + NH_4_]^+^	442.0	263.0	215.1	20	10-10	7.01
T2-G	[M + NH_4_]^+^	646.0	245.0	215.3	35	20-22	7.20
AFB1	[M + H]^+^	313.0	285.1	241.1	35	23-34	5.63
AFM1	[M + H[^+^	328.9	272.9	229.0	30	20-35	5.18
OTA	[M + H]^+^	404.0	238.9	220.8	35	20-32	9.23
ENN A1	[M + H]^+^	668.2	210.1	227.9	80	20-20	12.96
ENN A	[M + Na]^+^	704.5	350.1	232.2	35	48-48	13.12
ENN B	[M + H]^+^	640.1	213.8	527.2	80	22-21	12.69
ENN B1	[M + NH_4_]^+^	671.5	196.2	214.3	30	28-28	12.84
BEA	[M + H]^+^	784.1	244.0	262.1	80	25-30	12.81
FB2	[M + H]^+^	706.10	318.2	336.2	60	35-42	10.07
[^13^C_15_]-Deoxynivalenol	[M + H]^+^	312.0	263.0	245.0	20	10-10	4.37
[^13^C_17_]-Aflatoxin B1	[M + H]^+^	330.10	255.1	301.0	20	35-28	5.63
[^13^C_20_]-Ochratoxin A	[M + H]^+^	424.0	250.0	377.1	20	25-15	9.23
[^13^C_24_]-T2-toxin	[M + NH_4]_^+^	508.40	229.1	198.2	25	20-20	8.24
[^13^C_34_]-Fumonisin B1	[M + H]^+^	756.50	356.2	374.3	15	40-35	9.67
[^15^N_3_]-Enniatin B	[M + H]^+^	643.30	197.1	215.30	80	18-18	12.69

Note: *m*/*z* = mass-to-charge ratio; (a-b): collision energy for the quantifier (a) and qualifier ion (b), respectively.

## Supplementary Materials

The following are available online at https://www.mdpi.com/2072-6651/11/3/171/s1, Table S1: Overview of the theoretical accurate mass of the 24 mycotoxins and possible phase I and/or phase II metabolites, Figure S1: Extracted ion chromatogram of the first peak (4.09 min) in the trace of ZEN-glucuconide [*m*/*z* 493.1788] with HRMS spectrum, Table S2: Validation results for linearity (linear range, correlation coefficient (r) and goodness-of-fit coefficient (g)) and limit of quantification (LOQ) of 24 mycotoxins in pig feces, Table S3: Validation results for linearity (linear range, correlation coefficient (r) and goodness-of-fit coefficient (g)) and limit of quantification (LOQ) of 24 mycotoxins in pig urine, Table S4: Validation results for linearity (linear range, correlation coefficient (r) and goodness-of-fit coefficient (g)) and limit of quantification (LOQ) of 24 mycotoxins in broiler chicken plasma, Table S5: Validation results for linearity (linear range, correlation coefficient (r) and goodness-of-fit coefficient (g)) and limit of quantification (LOQ) of 25 mycotoxins in broiler chicken excreta, Table S6: Results of the within-day and between-day precision and accuracy experiments for 24 mycotoxins in pig feces, Table S7: Results of the within-day and between-day precision and accuracy experiments for 24 mycotoxins in pig urine, Table S8: Results of the within-day and between-day precision and accuracy experiments for 24 mycotoxins in broiler chicken plasma, Table S9: Results of the within-day and between-day precision and accuracy experiments for 25 mycotoxins in broiler chicken excreta, Figure S2: Concentration–time curves of zearalenone (ZEN) and deoxynivalenol (DON) in plasma after intra-gastric administration of DON (36 µg/kg bw) and ZEN (3 mg/kg bw) to pigs (*n* = 8). The mean + SD is shown, Figure S3: ZEN metabolites extracted ion chromatograms for a blank pig feces sample, Table S10: Results of signal suppression enhancement (SSE) end extraction recovery (RE) in pig plasma, urine and feces spiked at 10 ng/mL or 10 ng/g, Table S11: Results of signal suppression enhancement (SSE) end extraction recovery (RE) in broiler chicken plasma and excreta spiked at 10 ng/mL or 10 ng/g, Table S12: Chemical structure and pKa-values of the 26 mycotoxins.
