# Supplementary material for: Multi LC-MS/MS and LC-HRMS Methods for Determination of 24 Mycotoxins including Major Phase I and II Biomarker Metabolites in Biological Matrices from Pigs and Broiler Chickens
Source: Toxins (Basel). 2019 Mar 19;11(3):171. doi: 10.3390/toxins11030171 (PMC6468661; doi:10.3390/toxins11030171)
Supplement: Supplementary file 1 [file toxins-11-00171-s001.pdf]

Supplementary Materials: Multi LC-MS/MS and LC-HRMS Methods for Determination of 24 Mycotoxins including Major Phase I and II Biomarker Metabolites in Biological Matrices from Pigs and Broiler Chickens

Marianne Lauwers, Siegrid De Baere, Ben Letor, Michael Rychlik, Siska Croubels and Mathias Devreese

Table S1. Overview of the theoretical accurate mass of the 24 mycotoxins and possible phase I and/or phase II metabolites.

Name	Theoretical Accurate Mass (g/mol)	Name	Theoretical Accurate Mass (g/mol)
Deoxynivalenol	296.1260	$^{13}C_{15}$-Deoxynivalenol	311.1763
De-epoxy-deoxynivalenol	280.1311	$^{13}C_{17}$-Aflatoxin B1	329.1204
3/15-acetyldeoxynivalenol	338.1366	$^{13}C_{20}$-Ochratoxin A	423.1494
T2-toxin	466.2203	$^{13}C_{24}$-T2-toxin	490.3008
HT2-toxin	424.2097	$^{13}C_{34}$-Fumonisin B1	755.5025
Aflatoxin B1	312.0634	$^{15}N_{3}$-Enniatin B	642.4006
Aflatoxicol	314.0790	$^{13}C_{6}{}^{15}N$-Tenuazonic acid	207.1387
Aflatoxin M1	328.0538	$^{13}C_{18}$-Zearalenone	336.2071
Ochratoxin A	403.0823	Deoxynivalenol-glucuronide	472.1581
Enniatin A1	667.4408	De-epoxy-deoxynivalenol glucuronide	456.1632
Enniatin A	681.4564	Deoxynivalenol-sulphate	376.0828
Enniatin B	639.4095	Deoxynivalenol-di-sulphate	456.0396
Enniatin B1	653.4251	3/15-acetyl-deoxynivalenol-sulphate	418.0934
Beauvericin	783.4095	Ochratoxin alfa	256.0139
Fumonisin B2	705.3936	Zearalenone-glucuronide	494.1788
Tenuazonic acid	197.1052	Zearalenone-di-glucuronide	670.2109
Alternariol	258.0528	α/β-zearalenol- or zearalanone-glucuronide	496.1945
Alternariol methyl ether	272.0685	Zearalenone-sulphate	398.1035
Zearalenone	318.1467	α/β-zearalenol- or zearalanone-sulphate	400.1192
Zearalanone	320.1624	α/β-zearalenol-sulphate	402.1348
α-Zearalenol	320.1624	Tenuazonic acid-sulphate	277.0620
α-Zearalanol	322.1780	Alternariol-sulphate	338.0096
β-Zearalanol	322.1780	Alternariol-methyl ether sulphate	352.0253
β-Zearalenol	320.1624		

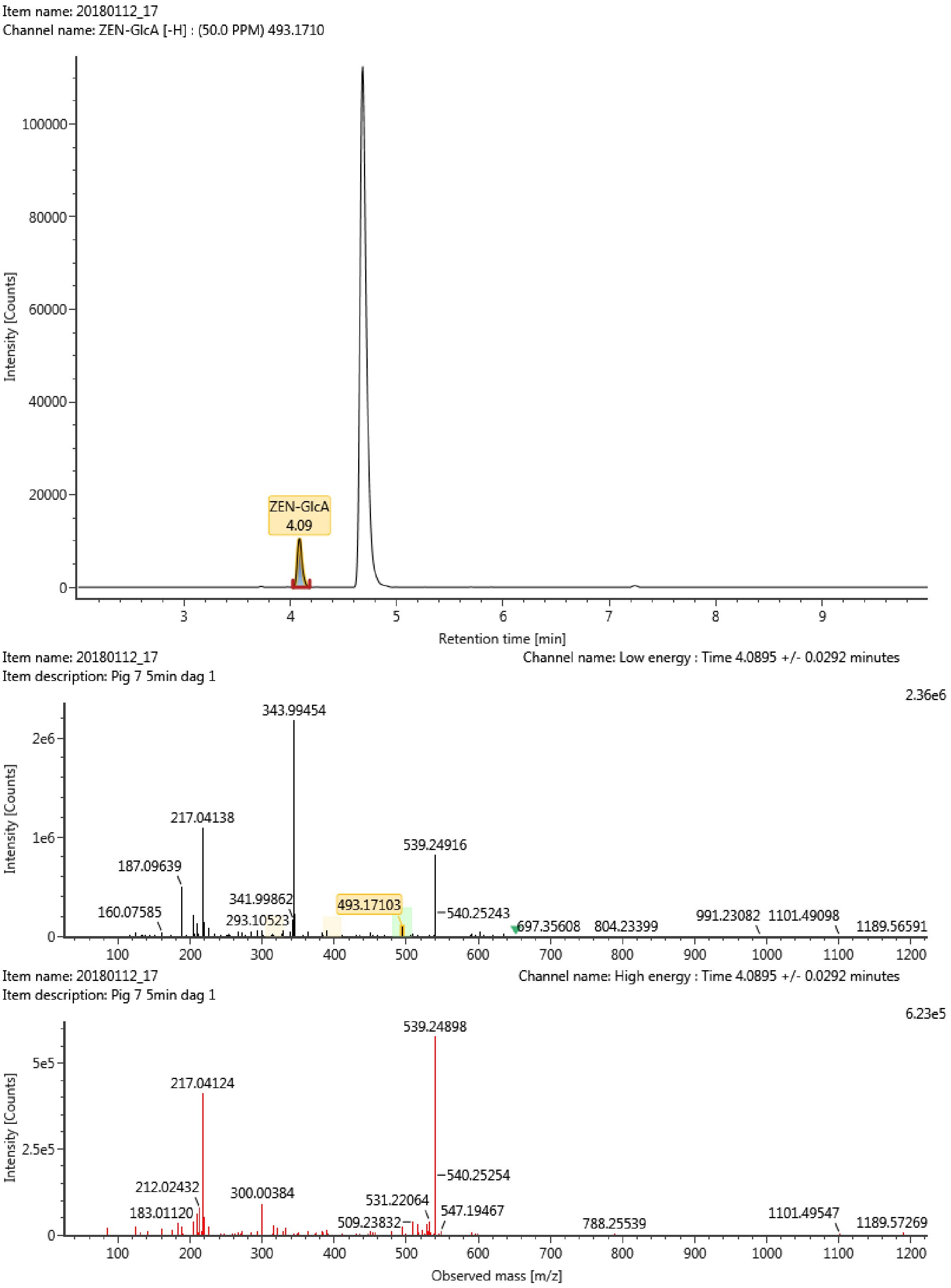

Figure S1. Extracted ion chromatogram of the first peak (4.09 min) in the trace of ZEN-glucuronide (ZEN-GlcA) [m/z 493.1788] with HRMS spectrum.

Table S2. Validation results for linearity (linear range, correlation coefficient (r) and goodness-of-fit coefficient (g)) and limit of quantification (LOQ) of 24 mycotoxins in pig faeces.

Analyte	Linearity (*n* = 3 Different Days)			LOQ (ng/g)
	Linear Range (ng/g)	R ± SD	g (%) ± SD	
ZEN	5–200	0.996 +/- 0.001	7.1 +/- 1.0	5.0
AZEL	1–200	0.996 +/- 0.001	15.7 +/- 4.3	1.0
AZAL	5–200	0.996 +/- 0.002	10.8 +/- 2.2	5.0
BZAL	1–200	0.996 +/- 0.002	9.0 +/- 3.5	1.0
BZEL	1–200	0.995 +/- 0.002	14.7 +/- 5.2	1.0
ZAN	1–200	0.997 +/- 0.003	11.5 +/- 4.2	1.0
TEA	1–200	0.999 +/- 0.000	18.7 +/- 0.9	1.0
AOH	5–200	0.997 +/- 0.001	8.9 +/- 3.1	5.0
AME	1–200	0.998 +/- 0.001	17.3 +/- 1.4	1.0
DON	5–200	0.994 +/- 0.003	14.1 +/- 4.4	5.0
DOM-1	5–200	0.997 +/- 0.000	8.8 +/- 3.1	5.0
3/15 ADON	1–200	0.998 +/- 0.001	13.4 +/- 4.2	1.0
T2	1–200	0.999 +/- 0.001	9.6 +/- 0.3	1.0
HT2	5–200	0.994 +/- 0.004	12.6 +/- 4.1	5.0
T2G	5–200	0.993 +/- 0.003	15.2+/- 5.3	5.0
AFB1	1–200	0.998 +/-0.002	10.5 +/- 4.6	1.0
AFM1	1–200	0.998 +/-0.000	7.9 +/- 4.2	1.0
OTA	1–200	0.997+/-0.001	8.9 +/- 2.5	1.0
ENN A1	1–200	0.997 +/-0.002	15.4 +/- 6.1	1.0
ENNA	1–100	0.996 +/-0.003	12.6 +/- 7.0	1.0
ENNB	1–200	0.997 +/-0.001	14.9 +/- 5.4	1.0
ENNB1	1–200	0.997 +/-0.002	15.2 +/- 5.1	1.0
BEA	1–200	0.999 +/-0.001	11.3 +/- 1.4	1.0

Table S3. Validation results for linearity (linear range, correlation coefficient (r) and goodness-of-fit coefficient (g)) and limit of quantification (LOQ) of 24 mycotoxins in pig urine.

**Analyte**	**Linearity (*n* = 3 Different Days)**			**LOQ (ng/mL)**
	**Linear Range (ng/mL)**	**R ± SD**	**g (%) ± SD**	
ZEN	1–200	0.999 +/- 0.000	7.5 +/- 4.8	1.0
AZEL	1–200	0.998 +/- 0.001	10.8 +/- 7.0	1.0
AZAL	1–200	0.998 +/- 0.002	11.8 +/- 6.6	1.0
BZAL	1–200	0.999 +/- 0.000	6.3 +/- 3.0	1.0
BZEL	1–200	0.997 +/- 0.001	12.2 +/- 2.1	1.0
ZAN	1–200	0.998 +/- 0.001	13.1 +/- 5.2	1.0
TEA	1–200	0.998 +/- 0.002	11.0 +/- 4.6	1.0
AOH	1–200	0.995+/- 0.003	16.8 +/- 3.2	1.0
AME	1–200	0.997+/- 0.001	10.2 +/- 2.3	1.0
DON	1–200	0.998 +/- 0.001	9.3 +/- 1.1	1.0
DOM-1	4–200	0.995 +/- 0.004	9.3 +/- 5.8	4.0
3/15 ADON	1–200	0.998 +/- 0.001	11.9 +/- 5.0	1.0
T2	1–200	0.999 +/- 0.000	9.7 +/- 3.7	1.0
HT2	1–200	0.995 +/- 0.003	14.1 +/- 5.2	1.0
T2G	2–200	0.996 +/- 0.002	17.0 +/- 1.9	2.0
AFB1	1–200	0.999 +/- 0.000	5.1 +/- 4.2	1.0
AFM1	1–100	0.997 +/- 0.004	10.5 +/- 7.8	1.0
OTA	1–200	0.999 +/- 0.001	8.7 +/- 1.2	1.0
ENN A1	1–200	0.999 +/- 0.001	11.2 +/- 1.9	1.0
ENNA	1–200	0.998 +/- 0.002	7.6 +/- 4.6	1.0
ENNB	1–200	0.999 +/- 0.000	3.5 +/- 2.7	1.0
ENNB1	1–50	0.999 +/- 0.001	5.9 +/- 3.0	1.0
BEA	1–100	0.999 +/- 0.000	11.2 +/- 5.3	1.0

Table S4. Validation results for linearity (linear range, correlation coefficient (r) and goodness-of-fit coefficient (g)) and limit of quantification (LOQ) of 24 mycotoxins in broiler chicken plasma.

Analyte	Linearity (*n* = 3 Different Days)			LOQ (ng/mL)
	Linear Range (ng/mL)	R ± SD	g (%) ± SD	
ZEN	1–200	0.998 +/- 0.001	8.0 +/- 6.4	1.0
AZEL	1–200	0.998 +/- 0.001	15.4 +/- 6.5	1.0
AZAL	1–200	0.997 +/- 0.003	12.0 +/- 5.2	1.0
BZAL	1–200	0.998 +/- 0.002	9.7 +/- 4.5	1.0
BZEL	1–200	0.998 +/-0.001	10.1 +/- 6.0	1.0
ZAN	1–200	0.998 +/-0.001	12.2 +/- 4.1	1.0
TEA	1–200	0.994+/-0.004	17.7 +/- 2.7	1.0
AOH	1–200	0.997 +/-0.002	13.6 +/- 5.8	1.0
AME	1–100	0.997 +/-0.002	15.6 +/- 5.6	1.0
DON	1–200	0.998 +/- 0.001	12.4 +/- 3.8	1.0
DOM-1	1–200	0.998 +/- 0.001	12.7 +/- 3.2	1.0
3/15 ADON	1–200	0.999 +/- 0.001	8.7 +/- 1.3	1.0
T2	1–200	0.997 +/- 0.003	7.7 +/- 3.7	1.0
HT2	1–200	0.999+/- 0.001	14.1+/- 8.0	1.0
T2G	5–200	0.996 +/- 0.004	9.0 +/- 5.0	5.0
AFB1	1–200	0.998+/- 0.002	6.3 +/- 4.3	1.0
AFM1	1–200	0.998 +/- 0.001	8.9+/- 5.0	1.0
OTA	1–200	0.998 +/- 0.002	12.7 +/- 3.5	1.0
ENN A1	1–200	0.999 +/- 0.000	7.9 +/- 1.0	1.0
ENNA	1–200	0.998+/- 0.001	16.2 +/- 4.3	1.0
ENNB	1–100	0.999 +/- 0.000	5.9 +/- 3.1	1.0
ENNB1	1–200	0.997 +/- 0.002	14.0 +/- 2.2	1.0
BEA	1–200	0.999 +/- 0.001	8.9 +/- 5.4	1.0

Table S5. Validation results for linearity (linear range, correlation coefficient (r) and goodness-of-fit coefficient (g)) and limit of quantification (LOQ) of 25 mycotoxins in broiler chicken excreta.

Analyte	Linearity (*n* = 3 Different Days)			LOQ (ng/g)
	Linear Range (ng/g)	R ± SD	g (%) ± SD	
ZEN	1–200	0.998 +/- 0.001	14.8 +/- 3.1	1.0
AZEL	1–200	0.998 +/- 0 .001	13.2 +/- 4.8	1.0
AZAL	1–200	0.996 +/- 0.003	15.1 +/- 6.7	1.0
BZAL	1–200	0.997 +/- 0.002	13.9 +/- 4.7	1.0
BZEL	1–200	0.996 +/- 0.003	14.8 +/- 1.8	1.0
ZAN	1–200	0.999 +/- 0.001	13.7 +/-4.7	1.0
TEA	1–200	0.998 +/- 0.001	12.4 +/- 4.6	1.0
AOH	1–200	0.997 +/- 0.001	14.9 +/- 4.3	1.0
AME	1–200	0.998 +/-0.000	13.5 +/-1.6	1.0
DON	1–200	0.998 +/- 0.001	13.5 +/- 0.9	1.0
DOM-1	1–200	0.998 +/- 0.001	13.5 +/- 3.9	1.0
3/15 ADON	1–200	0.995 +/- 0.003	14.5 +/- 5.2	1.0
T2	1–200	0.999 +/- 0.000	5.1 +/- 0.2	1.0
HT2	4–100	0.997 +/-0.001	16.5 +/- 3.0	4.0
T2G	2–200	0.998 +/-0.000	13.6 +/- 5.3	2.0
AFB1	1–200	0.997 +/- 0.002	12.1 +/- 6.9	1.0
AFM1	1–200	0.998 +/- 0.002	12.2 +/- 3.1	1.0
OTA	1–200	0.998 +/- 0.001	11.4 +/- 5.0	1.0
ENN A1	1–200	0.995 +/- 0.004	16.8 +/- 4.9	1.0
ENNA	1–200	0.995 +/- 0.003	15.9 +/- 1.4	1.0
ENNB	1–200	0.998 +/- 0.001	11.4 +/- 3.7	1.0
ENNB1	1–200	0.997 +/- 0.001	15.4 +/-3.8	1.0
BEA	1–200	0.995 +/- 0.002	15.4 +/-4.3	1.0
FB2	10–200	0.993 +/- 0.003	13.9+/-4.7	10

Table S6. Results of the within-day and between-day precision and accuracy experiments for 24 mycotoxins in pig faeces.

Analyte	Within-Day Precision and Accuracy (n = 6)						Between-Day Precision and Accuracy (n = 6)					
	Theoretical Concentration LOQ		Theoretical Concentration 10 ng/g		Theoretical Concentration 100 ng/g		Theoretical Concentration LOQ		Theoretical Concentration 10 ng/g		Theoretical Concentration 100 ng/g	
	Precision (RSD %)	Accuracy (%)	Precision (RSD %)	Accuracy (%)	Precision (RSD %)	Accuracy (%)	Precision (RSD%)	Accuracy (%)	Precision (RSD%)	Accuracy (%)	Precision (RSD %)	Accuracy (%)
ZEN	16.1	-21.2	3.7	-10.4	9.3	-3.2	21.2	-11.2	5.2	-10.4	9.7	-4.2
AZEL	6.9	1.6	7.2	-0.8	8.3	-1.7	28.0	4.0	6.1	1.3	6.8	-2.9
AZAL	3.3	10	6.5	2.5	1.3	-0.6	4.9	6.8	6.1	0.1	2.8	-2.4
BZAL	5.3	-17.6	7.9	-0.8	5.0	1.00	22.4	-10.1	12.1	-5.3	9.5	-2.0
BZEL	22.4	-29.0	2.8	-5.6	6.7	-4.7	37.9	-3.6	9.0	-0.5	5.3	-5.0
ZAN	21.6	-10.0	5.0	3.9	7.0	1.1	28.5	-19.5	9.0	0.6	7.8	2.9
TEA	11.0	18.2	6.7	-4.0	4.1	-7.6	27.7	12.5	8.5	-3.0	8.8	-3.6
AOH	14.1	-5.5	11.0	-3.4	5.9	-1.1	19.3	-5.9	11.9	-7.3	6.3	1.6
AME	19.4	-24.4	3.9	-9.2	8.4	4.6	39.2	-18.9	7.8	-8.5	8.3	4.0
DON	8.0	-8.7	5.6	-19.0	6.0	-18.9	7.7	-5.6	11.7	-12.4	12.7	-12.5
DOM-1	11.6	-4.0	9.7	-7.3	5.5	-2.8	10.5	-0.8	10.2	-7.0	7.3	-1.9
3/15 ADON	24.9	4.6	8.6	-13.6	7.9	-14.4	22.6	-0.8	7.5	-10.5	9.9	-8.6
T2	22.7	8.4	6.5	3.3	3.9	0.9	24.1	-0.5	6.2	2.8	4.1	3.1
HT2	7.7	1.1	7.0	1.0	7.2	-5.6	6.1	1.4	10.1	4.3	8.0	-2.0
T2G	22.2	-3.4	7.9	1.9	7.5	2.0	17.8	-0.1	8.6	-3.5	8.2	4.8
AFB1	18.6	-6.0	3.5	3.3	5.3	1.4	22.8	-9.9	5.6	1.6	4.1	1.8
AFM1	21.7	-1.6	2.2	-1.7	6.1	-4.0	29.9	6.0	1.9	-1.5	7.6	0.5
OTA	20.6	-8.8	4.4	-5.3	3.8	-3.8	18.8	-12.0	6.5	-4.1	5.3	-0.4
ENN A1	11.3	-10.7	6.6	9.9	7.0	6.5	25.3	0.5	11.4	3.2	10.9	-1.7
ENNA	21.5	-17.6	13.4	-5.8	5.7	-3.9	29.7	-22.8	15.7	0.8	13.0	1.5
ENNB	18.4	-7.2	6.6	6.0	4.3	2.3	21.7	4.5	7.3	5.4	15.4	-7.9
ENNB1	22.2	-9.1	5.7	-2.7	9.0	-8.6	30.8	-20.9	13.1	4.9	10.8	-6.1
BEA	20.8	-0.8	13.3	-7.9	8.9	1.0	25.6	15.8	17.0	1.4	12.4	-0.7

Table S7. Results of the within-day and between-day precision and accuracy experiments for 24 mycotoxins in pig urine.

Analyte	Within-Day Precision and Accuracy (*n* = 6)						Between-Day Precision and Accuracy (*n* = 6)					
	Theoretical Concentration LOQ		Theoretical Concentration 10 ng/mL		Theoretical Concentration 100 ng/mL		Theoretical Concentration LOQ		Theoretical Concentration 10 ng/mL		Theoretical Concentration 100 ng/mL	
	Precision (RSD %)	Accuracy (%)	Precision (RSD %)	Accuracy (%)	Precision (RSD %)	Accuracy (%)	Precision (RSD%)	Accuracy (%)	Precision (RSD%)	Accuracy (%)	Precision (RSD %)	Accuracy (%)
ZEN	12.0	-5.5	8.8	-5.8	2.4	-4.3	10.1	-6.2	7.2	-5.4	2.8	-3.1
AZEL	10.7	-12.6	11.6	-10.5	4.6	-7.4	12.6	-5.6	10.0	-8.1	6.7	-4.2
AZAL	9.8	14.3	12.2	-10.6	4.1	-15.1	7.9	12.1	9.7	-9.5	9.5	-8.8
BZAL	6.5	15.4	4.3	3.1	3.2	-1.3	6.5	15.4	4.3	3.1	3.2	-1.3
BZEL	13.5	-18.3	1.8	5.5	4.2	-7.5	12.1	-21.4	1.7	6.1	4.6	-5.8
ZAN	5.6	-4.0	2.0	7.9	6.4	-4.0	16.1	-8.5	3.4	7.1	5.2	-3.2
TEA	21.6	-17.1	6.7	-10.5	7.5	-0.2	18.9	-10.9	8.2	-7.5	7.1	1.0
AOH	24.8	-7.0	14.0	-8.5	7.2	2.6	32.1	-15.0	11.6	-7.9	10.8	-3.1
AME	21.3	-1.5	6.3	1.6	6.5	2.7	20.3	-5.0	17.0	-7.1	14.8	-4.8
DON	10.7	-14.3	4.8	-2.7	5.9	1.4	10.7	-14.3	4.8	-2.7	5.9	1.4
DOM-1	12.6	1.3	13.6	3.4	9.6	-2.9	9.0	2.1	12.0	-0.5	9.3	2.5
3/15ADON	17.4	14.5	4.9	3.5	5.5	-0.3	14.8	11.5	5.0	2.6	7.2	-1.7
T2	4.7	17.2	5.4	2.0	2.8	5.7	11.4	10.2	5.3	0	4.3	3.2
HT2	20.9	-10.0	8.6	-16.9	3.4	-7.4	23.1	-11.1	10.2	-17.4	3.4	-7.8
T2G	24.4	-8.4	5.1	4.7	4.8	-1.7	28.2	-14.1	12.6	-2.3	8.3	-5.0
AFB1	6.0	-8.0	3.0	2.0	1.7	-1.2	16.0	-12.8	3.4	1.5	1.9	-0.7
AFM1	7.0	19.2	7.6	4.9	5.6	-9.7	22.1	5.2	6.8	2.6	8.8	-8.4
OTA	10.7	-1.0	5.6	-2.8	4.7	2.9	9.5	2.1	4.4	-3.3	4.3	2.4
ENN A1	1.8	10.3	4.0	1.9	6.3	-5.2	5.6	6.8	3.3	1.7	5.5	-6.8
ENNA	7.5	-6.1	4.0	-15.1	9.4	-11.7	6.4	-5.1	4.9	-12.7	8.7	-11.1
ENNB	3.4	9.1	4.4	-0.4	8.2	-11.9	10.1	2.3	3.7	-1.2	6.4	-10.6
ENNB1	3.5	14.8	2.8	2.7	7.1	-1.2	6.0	12.6	3.1	2.4	5.8	-1.1
BEA	1.7	-1.7	7.5	-5.7	9.3	-8.2	6.8	-6.4	6.2	-4.7	11.1	-7.9

Table S8. Results of the within-day and between-day precision and accuracy experiments for 24 mycotoxins in broiler chicken plasma.

Analyte	Within-Day Precision and Accuracy ($n = 6$)						Between-Day Precision and Accuracy ($n = 6$)					
	Theoretical Concentration LOQ		Theoretical Concentration 10 ng/mL		Theoretical Concentration 100 ng/mL		Theoretical Concentration LOQ		Theoretical Concentration 10 ng/mL		Theoretical Concentration 100 ng/mL	
	Precision (RSD %)	Accuracy (%)	Precision (RSD %)	Accuracy (%)	Precision (RSD %)	Accuracy (%)	Precision (RSD%)	Accuracy (%)	Precision (RSD%)	Accuracy (%)	Precision (RSD %)	Accuracy (%)
ZEN	16.8	17.1	4.7	-6.5	4.0	-3.7	22.3	8.0	5.4	-5.2	4.9	-3.3
AZEL	10.2	-16.3	2.9	-15.7	9.5	-8.7	29.6	-6.9	10.6	-11.8	8.9	-4.1
AZAL	17.4	-34.3	5.5	-19.7	7.8	-9.4	36.0	-25.4	11.9	-13.3	8.0	-5.4
BZAL	14.6	-9.0	10.3	-19.5	8.6	-16.5	18.0	-0.2	13.8	-14.1	11.4	-10.2
BZEL	21.1	7.8	11.1	-15.0	8.8	-16.5	22.9	2.1	11.8	-9.9	13.6	-8.8
ZAN	21.2	-2.1	8.2	-8.1	8.1	-2.3	21.1	-8.2	6.5	-6.4	7.7	-3.1
TEA	22.3	-7.5	11.5	-19.7	8.0	-3.6	17.8	0.4	13.6	-13.3	11.4	-5.3
AOH	21.1	-3.3	9.6	-0.2	8.6	2.7	18.2	0.2	12.1	-5.4	8.7	0.5
AME	9.2	-9.3	4.5	8.7	8.9	6.8	19.7	-8.2	5.2	5.6	15.6	8.6
DON	22.7	14.0	9.4	1.8	4.5	-4.0	18.6	8.9	9.0	-0.6	4.7	-4.5
DOM-1	12.8	-9.4	6.9	2.9	5.4	-9.7	15.7	-14.5	8.1	2.7	6.8	-5.7
3/15ADON	3.9	19.6	13.2	2.7	6.4	-19.1	12.6	14.7	13.8	-2.9	13.2	-10.7
T2	3.5	2.2	7.7	-5.9	5.3	-2.0	6.7	-1.4	7.8	-3.9	7.9	-3.0
HT2	16.9	-6.0	4.0	-19.6	6.0	-18.8	19.4	-11.4	10.8	-14.5	12.1	-11.2
T2G	4.5	5.6	10.6	7.7	7.4	0.0	4.5	5.6	8.3	9.1	7.1	3.6
AFB1	4.0	7.0	3.8	-3.2	3.3	-3.2	10.1	0	4.4	-3.9	4.7	-3.8
AFM1	17.4	-28.4	6.1	-17.1	8.3	-19.9	17.4	-28.4	10.2	-13.4	12.0	-12.5
OTA	24.2	-7.2	3.8	-2.7	2.6	-5.0	23.8	-15.1	4.4	-4.8	3.1	-5.7
ENN A1	10.2	-30.7	12.7	0.8	8.9	-15.4	24.2	-17.3	10.6	-1.7	13.5	-7.5
ENNA	20.4	-15.1	9.7	2.0	8.0	-12.3	14.2	-14.1	8.1	1.2	9.8	-8.1
ENNB	2.1	5.6	3.5	-8.0	4.4	-2.6	7.7	2.1	3.2	-7.6	6.2	1.0
ENNB1	13.1	13.1	8.0	-2.9	8.9	0.5	24.6	-2.8	7.6	0.7	10.0	-1.9
BEA	3.8	3.3	10.9	-15.9	9.0	-11.1	16.0	-2.1	12.9	-8.9	12.6	-6.6

Table S9. Results of the within-day and between-day precision and accuracy experiments for 25 mycotoxins in broiler chicken excreta.

Analyte	Within-Day Precision and Accuracy (n = 6)						Between-Day Precision and Accuracy (n = 6)					
	Theoretical Concentration LOQ		Theoretical Concentration 10 ng/g		Theoretical Concentration 100 ng/g		Theoretical Concentration LOQ		Theoretical Concentration 10 ng/g		Theoretical Concentration 100 ng/g	
	Precision (RSD %)	Accuracy (%)	Precision (RSD %)	Accuracy (%)	Precision (RSD %)	Accuracy (%)	Precision (RSD%)	Accuracy (%)	Precision (RSD%)	Accuracy (%)	Precision (RSD %)	Accuracy (%)
ZEN	20.0	-23.3	6.1	8.5	2.8	7.3	15.7	-23.6	8.6	2.6	10.4	3.0
AZEL	11.1	-16.6	4.4	7.3	3.8	7.8	12.6	-17.1	15.5	-3.7	12.7	-0.2
AZAL	14.3	7.3	4.5	0.7	4.9	4.3	19.3	-1.1	8.7	-5.0	12.3	-2.7
BZAL	8.6	13.5	3.3	2.8	3.4	6.3	21.7	2.2	11.7	-4.3	13.3	-2.3
BZEL	16.6	-3.4	3.7	2.4	2.5	3.5	33.7	1.0	7.5	-0.5	6.2	-0.5
ZAN	13.1	-7.1	2.0	-0.1	2.5	6.5	19.0	-0.9	2.2	1.1	8.6	2.5
TEA	20.1	-15.9	8.4	-13.5	1.8	8.6	20.8	-16.8	9.5	-8.0	9.1	2.7
AOH	13.1	-20.4	13.7	3.1	3.2	8.2	16.9	-16.0	16.5	-4.0	14.4	0.5
AME	10.3	6.2	6.4	1.9	7.0	-14.6	16.6	-0.1	8.7	0	12.6	-10.2
DON	18.5	16.2	6.4	7.3	4.4	2.7	20.1	18.4	8.8	3.8	6.1	3.4
DOM-1	10.5	-2.2	3.3	7.2	2.0	8.0	27.4	-17.2	6.0	4.0	9.1	4.2
3/15ADON	9.3	7.0	12.5	-3.2	10.4	-14.3	15.4	-0.1	11.8	-1.8	14.9	-14.6
T2	6.9	-10.5	2.3	0.6	1.9	4.4	13.0	-10.8	4.1	-0.6	3.4	4.5
HT2	15.3	-13.6	7.0	-9.8	3.6	0.7	15.3	-13.6	15.0	-4.7	7.0	0.7
T2G	17.0	7.7	7.2	-1.7	7.0	7.7	13.3	8.7	6.4	-0.4	7.0	5.4
AFB1	10.7	-25	1.6	-1.2	2.4	1.5	13.4	-23.2	3.4	0.6	2.9	1.6
AFM1	21.0	-15.3	1.7	4.9	4.4	-10.6	14.2	-1.2	14.5	-7.7	19.0	-4.5
OTA	12.1	-4.4	6.3	-2.5	5.1	-2.2	44.7	-25.0	7.0	-0.8	8.9	-0.2
ENN A1	11.9	-30.8	2.6	4.8	1.0	5.5	23.9	-20.8	2.5	5.3	11.1	-0.4
ENNA	21.9	12.5	7.7	-17.4	6.2	-17.1	23.9	0.9	13.5	-10.5	9.4	-12.5
ENNB	20.0	-16.1	2.2	4.9	1.1	3.7	20.0	-14.2	2.4	5.5	8.8	0.6
ENNB1	8.0	-14.5	3.6	5.3	3.2	4.0	23.1	-20.9	4.7	4.4	13.4	-5.2
BEA	21.4	-8.5	1.9	8.2	2.1	8.8	43.7	-17.5	6.2	7.4	10.9	1.0
FB2	-	-	5.6	3.9	8.5	-0.4	-	-	4.7	3.6	7.9	0.4

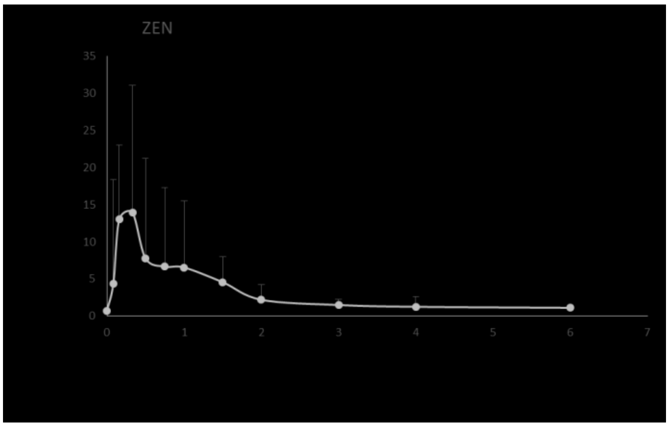

(a)

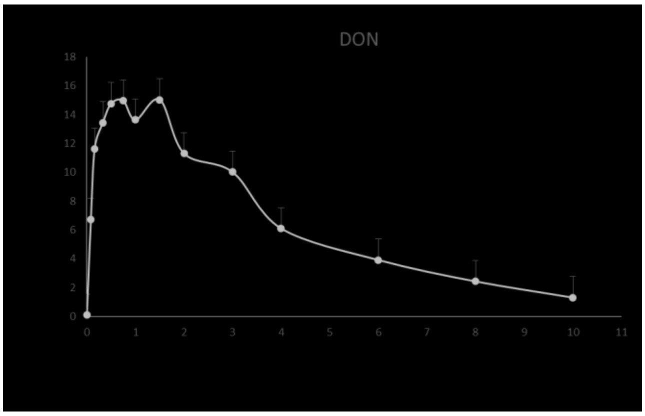

(b)

Figure S2. (**a**) Concentration-time curves of zearalenone (ZEN) in plasma after intragastric administration of ZEN (3 mg/kg bw) to pigs (*n* = 8). The mean + SD is shown. (**b**) Concentration-time curves of deoxynivalenol (DON) in plasma after intragastric administration of DON (36 μg/kg bw) to pigs (*n* = 8). The mean + SD is shown.

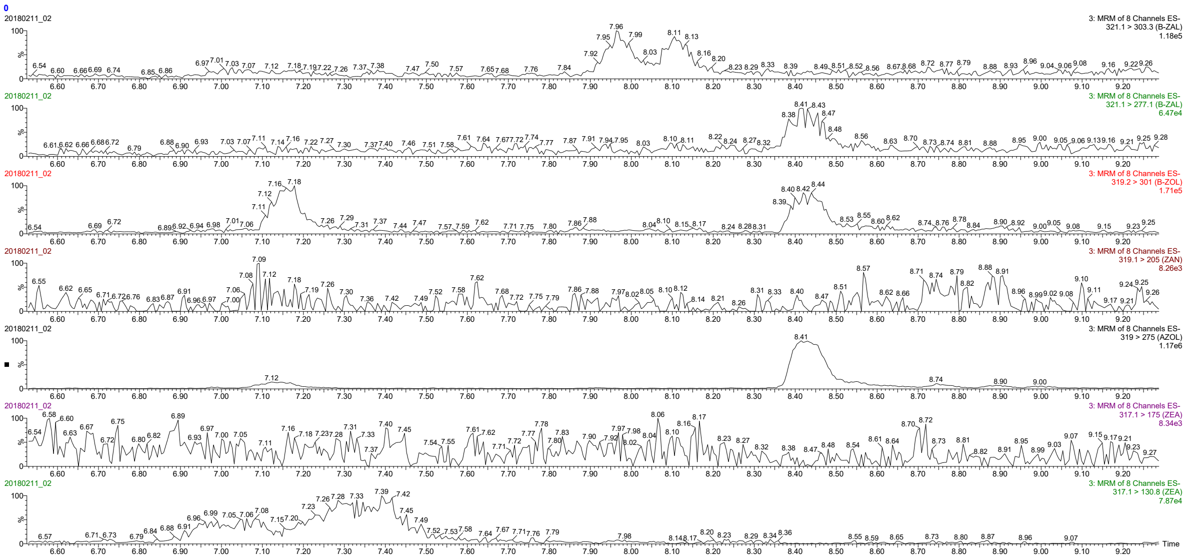

Figure S3. Zearalenone (ZEN) metabolites extracted ion chromatograms for a blank pig faeces sample.

Table S10. Results of signal suppression enhancement (SSE) end extraction recovery (RE) in pig plasma, urine and faeces spiked at 10 ng/mL or 10 ng/g.

Analyte	SSE (%)			RE (%)		
	**Pig Plasma**	**Pig Faeces**	**Pig Urine**	**Pig Plasma**	**Pig Faeces**	**Pig Urine**
ZEN	86.2	35.4	90.6	87.5	88.3	81.2
AZEL	74.7	22.4	84.8	88.1	93.4	80.5
AZAL	73.0	18.5	85.8	83.6	72.6	78.8
BZAL	74.4	30.5	85.1	83.0	60.5	79.9
BZEL	79.2	40.3	85.5	81.9	99.9	79.8
ZAN	86.5	30.3	90.6	83.5	66.7	79.5
TEA	96.3	12.4	52.4	72.9	56.4	62.6
AOH	114.9	13.5	11.1	87.4	88.5	212.8
AME	90.1	22.7	73.6	91.8	86.2	80.5
DON	40.4	27.5	79.5	95.4	266.1	67.3
DOM-1	42.6	23.7	5.2	96.3	131.0	69.6
3/15ADON	93.5	16.7	72.6	87.5	89.3	70.3
T2	83.3	82.4	90.5	96.2	60.1	56.2
HT2	61.7	62.8	100.4	110.6	93.4	75.1
T2G	71.5	66.4	107.6	87.3	41.2	74.4
AFB1	87.4	35.	72.4	79.5	59.3	60.0
AFM1	50.1	35.3	79.3	85.9	61.4	62.0
OTA	101.3	68.1	106.3	131.5	54.6	73.4
ENN A1	62.1	78.6	81.3	79.2	48.2	80.3
ENNA	66.8	304.5	80.2	132.2	61.9	79.6
ENNB	50.9	61.9	83.7	90.0	68.6	79.5
ENNB1	45.5	78.2	82.8	86.8	46.5	73.9
BEA	64.3	108.9	89.5	92.1	69.1	78.9

Table S11. Results of signal suppression enhancement (SSE) end extraction recovery (RE) in broiler chicken plasma and excreta spiked at 10 ng/mL or 10 ng/g.

Analyte	SSE (%)		RE (%)	
	Chicken Plasma	Chicken Excreta	Chicken Plasma	Chicken Excreta
ZEN	76.8	146.2	66.0.	62.9
AZEL	65.7	66.6	60.1	24.8
AZAL	100.0	89.3	61.8	65.6
BZAL	64.5	105.8	64.1	65.4
BZEL	67.7	120.5	62.8	63.5
ZAN	79.3	146.9	59.8	52.7
TEA	121.9	21.0	63.8	64.4
AOH	99.9	50.8	57.3	43.6
AME	81.3	14.4	61.2	52.3
DON	34.8	52.3	72.2	76.0
DOM-1	41.6	41.5	71.4	90.5
3/15ADON	53.5	67.5	82.0	84.6
T2	110.8	89.9	73.4	70.0
HT2	106.2	157.1	92.2	53.8
T2G	112.6	85.2	63.5	57.3
AFB1	93.2	126.7	51.6	63.7
AFM1	72.3	359.4	67.9	15.7
OTA	107.6	58.2	90.9	68.1
ENN A1	135.5	90.3	66.2	70.2
ENNA	73.9	821.6	87.5	54.8
ENNB	78.4	73.1	70.0	80.1
ENNB1	94.9	70.4	70.2	93.7
BEA	92.4	80.7	70.0	73.6

Table S12. Chemical structure and pKa-values of the 26 mycotoxins.

**Analyte**	**Chemical Structure**	**pKa and Log P or log D Value at pH 3 /pH 7**	**Analyte**	**Chemical Structure**	**pKa and Log P or log D Value at pH 3 / pH 7**
ZEN	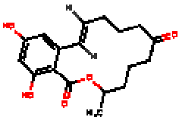<br>$C_{18}H_{22}O_5$<br>Acidic	pKa: 7.41 ± 0.4<br>Log P: 3.83 / 3.72	T2	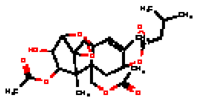<br>$C_{24}H_{34}O_9$<br>Neutral	pKa: 13.23 ± 0.7<br>Log P: 2.25 / 2.25
AZEL	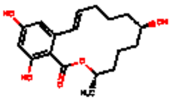<br>$C_{18}H_{24}O_5$<br>Acidic	pKa: 7.61± 0.6<br>Log P: 4.17 / 4.17	HT2	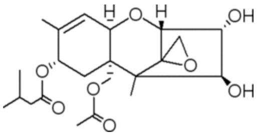<br>$C_{22}H_{32}O_8$<br>Neutral	pKa: 13.26 ± 0.7<br>Log P: 2.27 / 2.27
AZAL	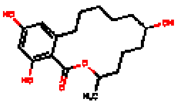<br>$C_{18}H_{26}O_5$<br>Acidic	pKa: 8.6 (strongest acid)*<br>Log P: ± 4*	T2G	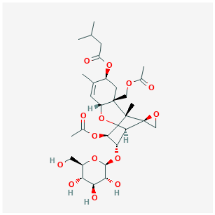<br>$C_{30}H_{44}O_{14}$	pKa: /<br>Log P: /
BZAL	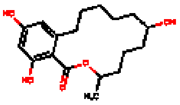<br>$C_{18}H_{26}O_5$<br>Acidic	pKa: 8.6 (strongest acid)*<br>Log P: ± 4*	AFB1	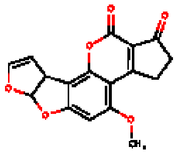<br>$C_{17}H_{12}O_6$<br>Neutral	pKa: 17.79 (strongest acid)*<br>Log P: 0.45
BZEL	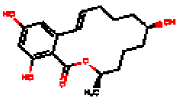<br>$C_{18}H_{24}O_5$	pKa: 7.61 ± 0.6<br>Log P: 4.17 / 4.17	AFM1	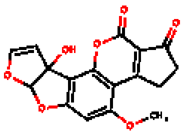<br>$C_{17}H_{12}O_7$	pKa: 11.42 (strongest acid)*<br>Log P: - 0.35

	Acidic			Neutral	
ZAN	$C_{18}H_{24}O_5$<br>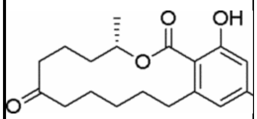	pKa: /<br>Log P: /	OTA	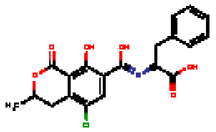<br>$C_{20}H_{18}ClNO_6$<br>Amphoteric	pKa: 3.29 ± 0.1 /-2.20 ± 0.4<br>log P: 4.41 / 1.10
TEA	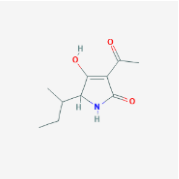<br>$C_{10}H_{15}NO_3$<br>Amphoteric	pKa: 4.5 ± 1 /-2.70 ± 0.7<br>Log P: 0.8 / -1.65	ENN A1	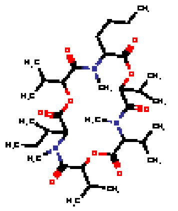<br>$C_{35}H_{61}N_3O_9$<br>Basic	pKa: -1.44 ± 0.7<br>Log P: 4.11 / 4.11
AOH	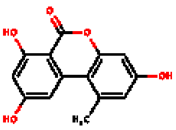<br>$C_{14}H_{10}O_5$<br>Acidic	pKa: 7.16 ± 0.20<br>Log P: 3.3 / 3.06	ENNA	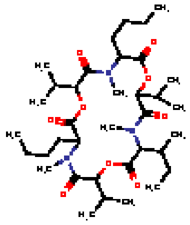<br>$C_{36}H_{63}N_3O_9$<br>Basic	pKa: -0.96 ± 0.7<br>Log P: 4.64 / 4.64
AME	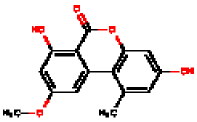<br>$C_{15}H_{12}O_5$<br>Acidic	pKa: 6.99 ± 0.20<br>Log P: 3.93 / 3.62	ENNB	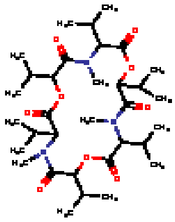<br>$C_{33}H_{57}N_3O_9$<br>Basic	pKa: -1.08 ± 0.7<br>Log P: 3.05 / 3.05

DON	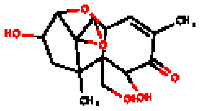<br>$C_{15}H_{20}O_6$<br>Neutral	pKa: 11.91 ± 0.7<br>Log P: -1.41 / -1.41	ENNB1	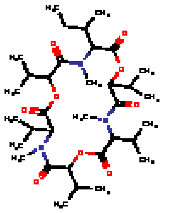<br>$C_{34}H_{59}N_3O_9$<br>Basic	pKa: -1.45 ± 0.7<br>Log P: 3.58 / 3.58
DOM-1	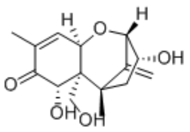<br>$C_{15}H_{20}O_5$<br>Neutral**	pKa: /<br>Log P: -0.71<br>(estimate)**	BEA	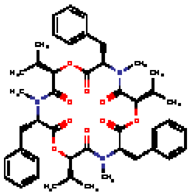<br>$C_{45}H_{57}N_3O_9$<br>Basic	pKa: 18.8 (strongest acid)*<br>Log P: ± 5.5 *
15-ADON	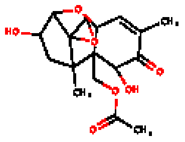<br>$C_{17}H_{22}O_7$<br>Neutral*	pKa: 12 (strongest acid) *<br>Log P: -0.54 *	FB1	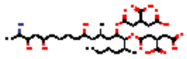<br>$C_{34}H_{59}NO_{15}$<br>Amphoteric	pKa: 3.64 ± 0.23 / 9.24 ± 0.39<br>Log P: - 0.61/-3.23
3-ADON	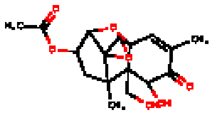<br>$C_{17}H_{22}O_7$<br>Neutral	pKa 11.84 ± 0.7<br>Log P: -0.76 / -0.76	FB2	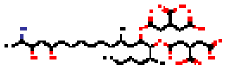<br>$C_{34}H_{59}NO_{14}$<br>Amphoteric	pKa: 3.64 ± 0.23 / 9.25 ± 0.39<br>Log P: 1.58 / -1.04

Source: E. Apfelthaler et al. [2] and **Toxin and Toxin Target Database (T3DB)* [1] **Pubchem. / = not found; Acidic= acidic solute; Basic= basic solute; Neutral= neutral solute; Amphoteric = Amphoteric solute.
